# Objective classification and scoring of movement deficiencies in patients with anterior cruciate ligament reconstruction

**DOI:** 10.1371/journal.pone.0206024

**Published:** 2019-07-23

**Authors:** Chris Richter, Enda King, Siobhan Strike, Andrew Franklyn-Miller

**Affiliations:** 1 Sports Medicine, Sports Surgery Clinic, Dublin, Ireland; 2 Department of Life Sciences, University of Roehampton, London, United Kingdom; 3 Centre for Health, Exercise and Sports Medicine, University of Melbourne, Melbourne, Australia; University of Rome, ITALY

## Abstract

Motion analysis systems are widely employed to identify movement deficiencies—e.g. patterns that potentially increase the risk of injury or inhibit performance. However, findings across studies are often conflicting in respect to what a movement deficiency is or the magnitude of association to a specific injury. This study tests the information content within movement data using a data driven framework that was taught to classify movement data into the classes: NORM, ACL^OP^ and ACL^NO OP^, without the input of expert knowledge. The NORM class was presented by 62 subjects (124 NORM limbs), while 156 subjects with ACL reconstruction represented the ACL^OP^ and ACL^NO OP^ class (156 limbs each class). Movement data from jumping, hopping and change of direction exercises were examined, using a variety of machine learning techniques. A stratified shuffle split cross-validation was used to obtain a measure of expected accuracy for each step within the analysis. Classification accuracies (from best performing classifiers) ranged from 52 to 81%, using up to 5 features. The exercise with the highest classification accuracy was the double leg drop jump (DLDJ; 81%), the highest classification accuracy when considering only the NORM class was observed in the single leg hop (81%), while the DLDJ demonstrated the highest classification accuracy when considering only for the ACL^OP^ and ACL^NO OP^ class (84%). These classification accuracies demonstrate that biomechanical data contains valuable information and that it is possible to differentiate normal from rehabilitating movement patterns. Further, findings highlight that a few features contain most of the information, that it is important to seek to understand what a classification model has learned, that symmetry measures are important, that exercises capture different qualities and that not all subjects within a normative cohort utilise ‘true’ normative movement patterns (only 27 to 71%).

## Introduction

Motion analysis systems are widely employed within both universities and clinical facilities, to explore the association between movement patterns and athletic performance or risk of injury, which is of great interest to coaches, physiotherapists and other medical professionals. However, while there are many studies that have examined movements using kinematic and kinetic measurements seeking to identify features (e.g. maximum knee flexion) that might be related to injury, there are no well established evidence-based guidelines that state what movement deficiencies are, what normative ranges of variability are or what a “normal” movement looks like. Additionally, there is little agreement as to which, if any, exercises or movement can expose patterns that could lead to injury or what properties an exercise should hold (single or double leg, movement in one, two or three planes, ecological validity and so on). For example, both Hewett et al., [1] and Krosshaug et al., [2] examined features extracted from a double leg drop jump (DLDJ) to assess their ability to predict the risk of sustaining an anterior cruciate ligament (ACL) injury. Both studies performed a prospective analysis on a large population ([1] = 205; [2] = 782) of female athletes participating in field sports. While Hewett et al., [1] concluded that specific features (describing knee motion and knee loading) were very sensitive in predicting ACL injury, Krosshaug et al., [2] reported poor prediction abilities. The conflict in findings between movement analysis studies, provoked Bahr [3] to state in a recent review: “To date, there is no screening test available to predict sports injuries with adequate test properties and no intervention study providing evidence in support for screening for injury risk”.

A possible source of conflicting conclusions is the way features are extracted. When describing a movement, studies often extract features based on prior knowledge (previous research and / or personal and clinical experience) or post hoc analysis to reduce dimensionality within the highly multivariate datasets, across joints and time. These features are then used to compare the magnitude or timing between groups [3] assuming that the extracted features capture the underlying function of a signal. While discrete points can be helpful in understanding movements, the selection of discrete points has the potential to: discard important information [4, 5], to compare features that present unrelated neuromuscular capacities [6] and to encourage fishing for significance—e.g. non-trivially biased non-directed hypothesis testing [7]. Due to the apparent limitations in discrete point selection, other analyses have been introduced in recent years—statistical parametric mapping [7], (functional) principal component analysis [8–10], analysis of characterising phases [6], point by point manner testing [11] and other techniques [3, 10, 12–14] to improve the analysis of movement patterns.

Another possible source for conflicting conclusions is the way extracted features are compared across groups. Comparisons are often made using statistical significance, and conclusions are developed by inferring properties about a population by testing hypotheses and deriving estimates by probability values (p-values). While, p values are useful they are frequently criticised [15–20] due to the possibility of committing type 1 and 2 errors, that any difference can be statistically significant—with large enough sample size [18]—that p values do not provide statistical precision and that conclusions do not account for multiple movement strategies (subgroups) within a dataset. As such, injury related features can be masked during an analysis. Differences in movement strategies within a dataset may be caused by differences in anthropometric measures as well as training, sporting and injury history and there is growing evidence for different movement strategies across individuals [21–31].

When examining a human movement, the method of data analysis chosen needs to contend with a complex and multivariate system and any analysis using an inference test may not help to progress the understanding of movement as it does not account for differences in movement strategy or the interrelationship of segments. More suitable methods for movement analysis might be a data driven machine learning techniques that do not require expert knowledge, which have gained popularity in biomechanics and other fields as they have demonstrated an enhanced ability to understand complex and multivariate systems—see Barton et al., [14], Halilaj et al., [32], Rajpurkar et al., [33] or Figueiredo et al. [34]. While studies do not frequently use machine learning techniques in sport-biomechanics, machine learning techniques have been used in gait studies to classify movement data into pathological and able-bodied [35], to detect / judge severity of diseases [36] or to predict surgical / therapy outcomes [37, 38]. For the interested reader Figueiredo et al. [34] reviewed experiments that used machine learning in gait-biomechanics, while Halilaj et al. [32] reviewed the usage of machine learning technique in human movement biomechanics stating best practices and common pitfalls. One of the few studies within sports-biomechanics that used a data-driven supervised learning approach to classify the skill level of athletes (novice and elite) using biomechanical data is Ross et al., [10] and reported an accuracy of 70 to 80%. However, machine learning techniques have not been applied to differentiate movement patterns between non-injured and rehabilitating athletes (e.g. after ACL reconstruction). As such it is not known a) what the most appropriate machine learning technique for such a classification task is, b) what classification accuracies one could expect or c) if movement patterns differ on a group level (e.g. non-injured vs. rehabilitating) or limb level (normal limb vs. operated limb and limb contralateral to operated limb) as movement pattern difference might by driven by anatomical/morphological differences, which could affect both legs or just one limb in rehabilitating cohorts.

The aim of this study was to develop and test a data driven framework (feature generation based on no expert or prior knowledge) to classify movement patterns of normal and rehabilitating athletes using only biomechanical data.

## Materials and methods

### Subjects

This study used a cohort of males recovering from ACL reconstruction as the rehabilitating cohort (ACL reconstruction should have altered kinematics and neuromuscular properties within the operated knee [39]) and a cohort of healthy males as control / normal group. The ACL group (n = 156) was recruited from the caseload of two orthopaedic surgeons who specialise in knee surgery between January 2014 and December 2016. Inclusion criteria were: biomechanical assessment approximately 9 months after ACL reconstruction, intention of returning to full participation in multi-directional sport after surgery, bone patellar tendon bone graft or hamstring graft from the ipsilateral side, gender (male) and an age between 18 and 35. Exclusion criteria were: multiple or previous ACL reconstructions and any meniscal repairs. The control group (n = 62) contained males that participated in multi-directional sport (i.e. Gaelic Football, Soccer, Hurling, Rugby Union) that were free of injury in the 3 months prior to testing, had no previous knee surgery and were between 18 and 35 years of age. The study received ethical approval from Sports Surgery Clinic Hospital Ethics committee (25AFM010) and was registered on clinicaltrials.gov (NCT02771548). The examined data were fully anonymised and all subjects provided informed written consent to have their data used for research.

The ACL group had an average age of 24.8 ± 4.8 years was 180 ± 8 cm tall and had a body mass of 84 ± 15.2 kg. The control group had an average age of 24.8 ± 4.2 years was 183 ± 6 cm tall and had a body mass of 82 ± 8.9 kg.

### Data capture and pre-processing

The testing took place in the motion analysis laboratory using an eight-camera motion analysis system (200Hz; Bonita-B10, Vicon, UK), synchronised with two force platforms (1000Hz BP400600, AMTI, USA). Before data collection, all subjects undertook a standardised warm-up and wore their own athletic footwear with 24 reflective markers secured to the shoe or to the skin using tape, at bony landmarks according to the Plug-in-Gait marker set. Three trials of each limb for the following seven exercises were captured: double leg (DL) countermovement jump (CMJ; [40]), single leg (SL) CMJ [40], DL drop jump (DJ; [40]), SLDJ [40], Hurdle Hop (HuHo; [40]), SL Hop (SLHop; [40]), planned change of direction (CoDP [41]) and unplanned change of direction (CoDU [41]). Marker and force data were low-pass filtered using a fourth-order Butterworth filter [42]—before computing kinematic and kinetic measures using Nexus (1.8.5; Vicon, UK). Data pre-processing (gap filling and waveform screening) was performed on examined biomechanical measures using a custom developed MATLAB program (R2015a, MathWorks Inc., USA). All kinetic variables were normalised to body mass. The start and end point of an exercise was defined using the force trace or a combination of center of mass (CoM) power and force trace [40, 41] and all measures were landmark registered using a dynamic time warping process [43] to align the end of the eccentric phase across the all curves [40, 41]. Only the maximum trial of the captured (based in maximal jump height and shortest contact time) was chosen for data analysis.

### The framework

The steps taken during data analysis can be described as follows: feature engineering, selection of a supervised learning technique, feature selection and final validation of minimal feature model. The performance of any generated classification model described in this study is the average classification accuracy of a 100 split stratified shuffle split cross-validation. A shuffle spilt cross-validation was used in preference to a k-fold cross validation because, although information leaking can occur between the splits, it allows the re-use of subjects that have been assigned previously to the trainings group and hence a number of splits and greater sample size during the training / fitting of the prediction model. The description of the generation of the framework is based on a single exercise and was done for every exercise separately.

During the performed splits, false and correct classifications were recorded to allow the generation of a confusion matrix for every model generated on a group (e.g. ACL and NORM), limb (class: ACL^OP^, ACL^NO OP^ and NORM) and subject level. This information is essential when examining the prediction model, in respect to errors (confusion or misclassifications) made and can also help in understanding the examined data—e.g. do features differ on a group or limb level (misclassifications of class but not group level), do some subjects behave differently than others within a class (misclassification of specific subjects) or if one model made misclassifications another did not (could to models complemented each other). In addition to recording false and correct classifications, during every split a “guess prediction” was made with each trial having a 1 in 3 chance of belonging to the class: ACL^OP^, ACL^NO OP^ or NORM. The best guess, the highest guess classification accuracy observed within splits, was then used as a threshold to judge the meaningfulness of the generated models. A classification model was defined meaningful if the lowest observed accuracy during the splits was greater than the best guess.

This study evaluated the created models on a number of levels: the number of features selected for inclusion (3 levels: all features, 20 features, minimal feature), the machine learning technique (7 widely used techniques were applied) and movement type (8 movement frequently used to assess the rehabilitation status of athletes with ACL and to predict future injury). Except of the minimal feature model, the evaluation was performed by assessing the average classification accuracy. Evaluation of the minimal feature model was based on average classification accuracy, classification errors and sensitivity. Other classification performance measures e.g. area under the curve, precision, recall, f1-score or specificity would very likely have resulted in different models and performance rankings.

#### Feature engineering

Traditional analyses, which use discrete measures, are dependent on the selection of features. To enable a data-driven feature engineering that does not require expert knowledge, this study reduced dimensionality of commonly examined motion capture time-series by examining variability within the time-series across examined in 100 random shuffle splits using analysis of characterising phases [6]. This information was then used to reduce a time-series to just a few features that describe the variability of the signal across all participants. This process was performed on each examined time series separately. The following 82 time series were examined separately: ground reaction force (GRF; x, y, z), GRF impulse (x, y, z), center of mass (CoM) velocity (xy, xyz, z), CoM power (x, y, z), CoM in pelvis, hip, knee and ankle (x, y, z) as well as joint angles of the ankle, knee, hip, pelvis, thorax and thorax on pelvis in sagittal, frontal and transversal planes, joint angular velocities of the ankle, knee, hip, pelvis, thorax and thorax on pelvis in sagittal, frontal and transversal plane, joint powers, moments, work and impulse of ankle, knee, hip and pelvis in sagittal, frontal and transversal plane, time and the rotation foot angle to pelvis.

**Fig 1:**
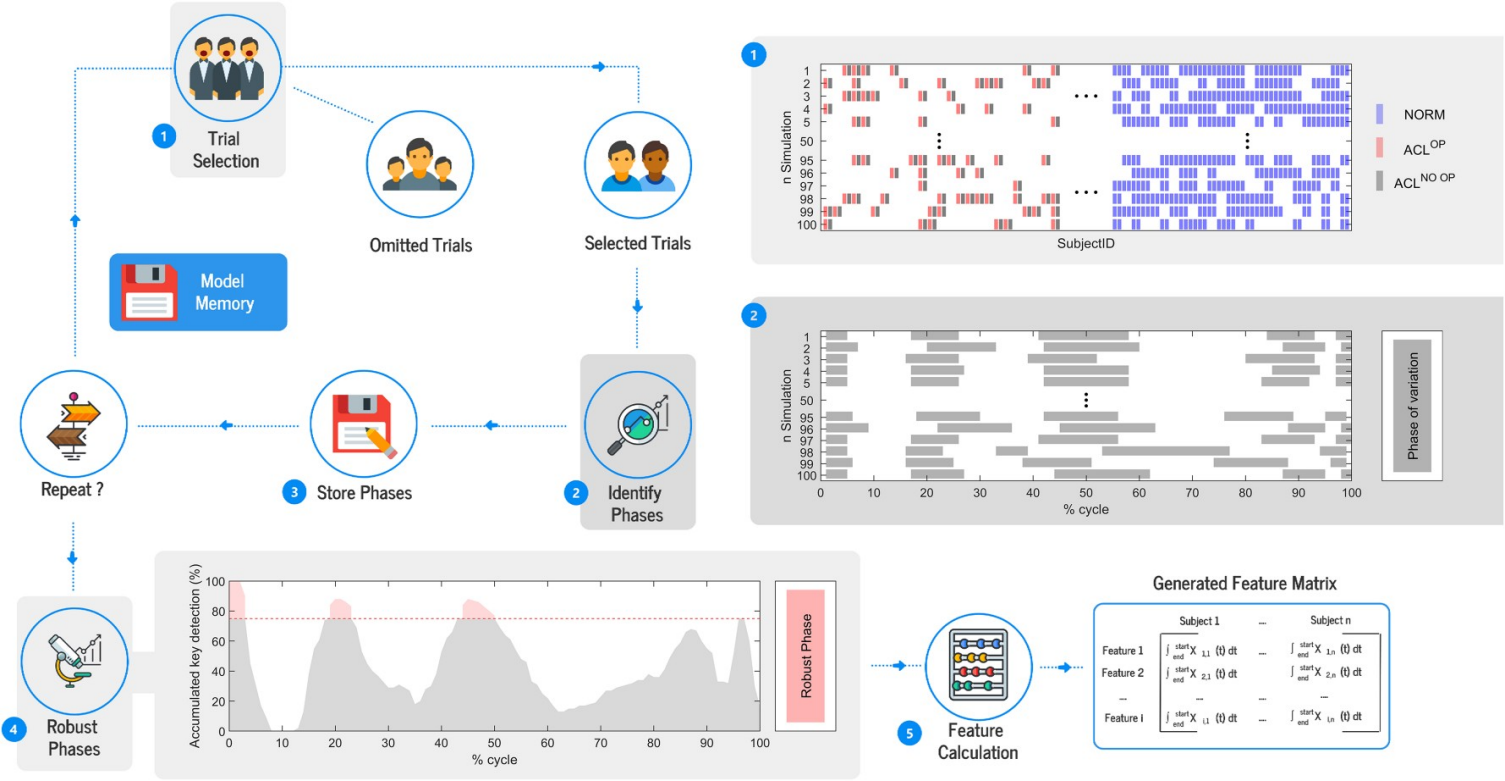
Illustration of the work-flow during the features engineering.

Before performing the stratified shuffle splits, a matrix (memory matrix) was defined for every examined signal consisting of 101 rows (corresponding to length of the time-series) and 100 columns (corresponding to the number of splits) storing “0” values. The first step within every split was the random selection of 50 subjects from the ACL and NORM cohort to create a dataset of 200 trials—containing 50 ACL^OP^ limbs, ACL^NO OP^ limbs and 2x50 NORM limbs (Fig 1—step 1). Analysis of characterising phases (described in [6]) was then used to identify phases of variation within the created dataset. For every time point that was within a detected phase of variation, the default “0” value was changed to “1” for the corresponding row and column in the memory matrix. For example, if the described process identified the time points from 5 to 15 within a time series to be a phase of variation in the 7^th^ split, then the value of row 5 to 15 in column 7 in the memory matrix was changed to “1” (Fig 1—step 2).

After completing the 100 splits, the sum of the values in the memory matrix was computed along the split axis generating a 101x1 vector that stored the accumulated appearance as a phase of variation for each frame. A value of “0” indicates that this time point was never within a phase of variation, while the value 84 indicates that a time point was 84 times within a phase of variation (Fig 1—step 4). Every collection of time points that occurred in 75% of the splits and spanned at least 5% of the movement cycle was considered robust phase of variation and used for the feature engineering. The investigators used a 75% threshold, instead of 95% [44], to maintain more information—i.e. allowing more phases to be classified as robust phases of variation. Features describing a phase were calculated as the average value of a phase and stored into a feature matrix that contained *i* rows / features (*i* is the number of detected phases of variation) and *n* = 436 columns / observations (156 operated limbs [class: ACL^OP^], 156 limb contralateral to the operated limb [class: ACL^NO OP^] and 124 control limbs [class: NORM]). For every double leg exercise, symmetry was also calculated as *limb* – *contralateral limb*—e.g. *left* – *right* for left leg trials or *right* – *left* for right leg trials.

During the feature engineering the data examined for each exercise generated between 97 and 186 features for each of the 436 observations (Table 1). The detailed description of results of this step is given in S1 Appendix—Detected Phases. These features were used within the following steps as input features during the fitting (training) and validation (testing) of every generated model.

**Table 1:** Number of extracted features that were used as input features during fitting (training) and validation (testing) of the supervised learning techniques.

	SLCMJ	DLCMJ	SLDJ	DLDJ	HuHo	SLHop	CoDP	CoPU
i features	140	176	97	186	119	157	132	97
n trials	436	436	436	436	436	436	436	436

#### Selection of supervised learning technique

The second step in the model generation was the identification of the most appropriate machine learning technique. This process was done because the selection of the best learning technique can be challenging as there are multiple techniques and each technique has different abilities to learn relationships between the to be predicted classes and input features [45, 46]. This study examined only the ability of techniques included within the statistical toolbox of Matlab (R2015a, MathWorks Inc., USA): a decision tree (fitctree), an ensemble of decision trees (n trees = 50; TreeBagger), a discriminant analysis model (fitcdiscr), a naive Bayesian classifier (fitcnb), a k-nearest neighbour model (k = 5; fitcknn), a multi class model for support vector machines (fitcecoc), a linear regression model (mnrfit; in stepwise forward) and a neural network (patternnet). No grid search was performed at any time and is it very likely that accuracies in better-tuned models will outperform the presented findings. The random assignment of observations into training and testing dataset, during a split, was performed on athlete level to prevent data leakage from training into testing data—e.g. if a subject was selected to belong to the training dataset both the right and left limb were used within the training. Each learning technique was tested separately using raw values and normalised values (z-scores) of the feature matrix.

**Fig 2:**
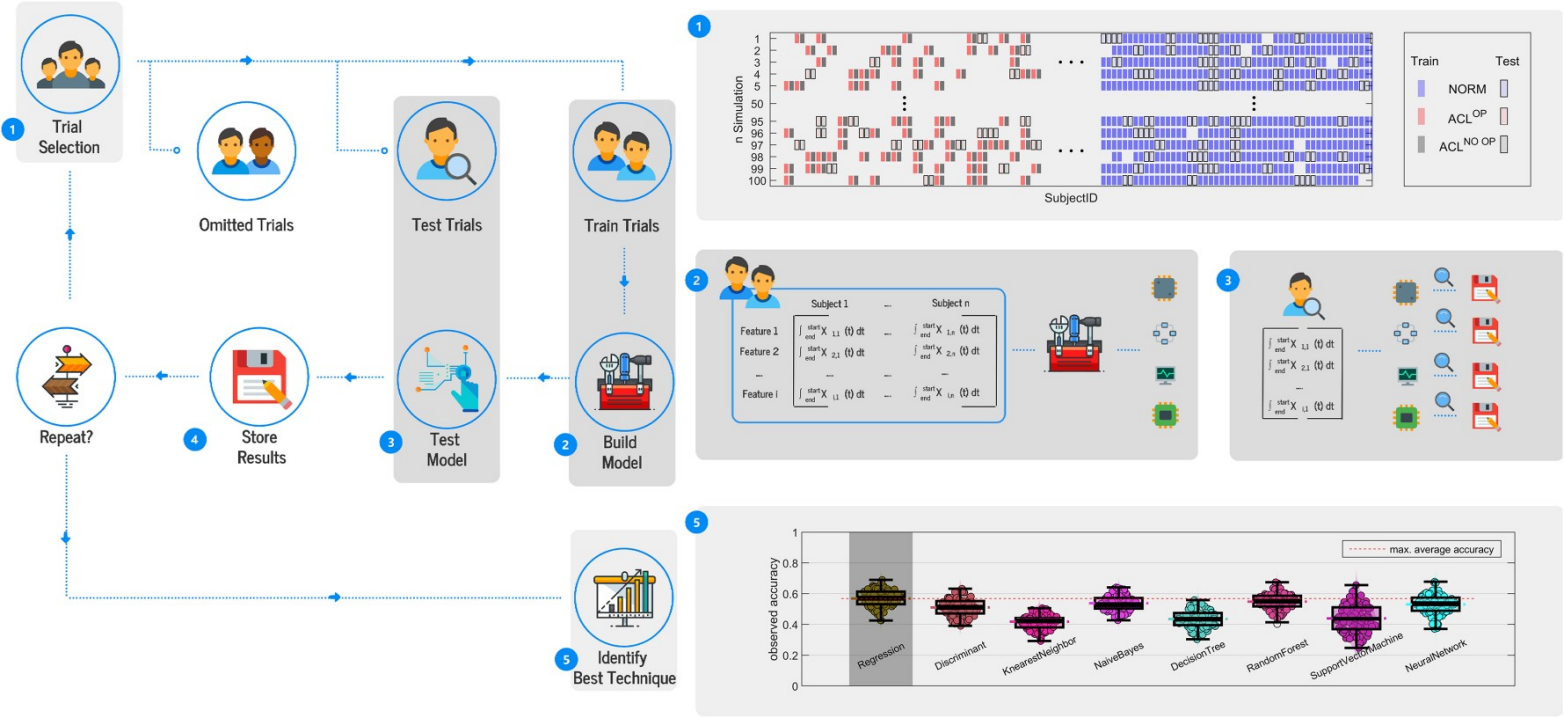
Illustration of the workflow to identify the most suitable learning technique.

During a split, the feature matrix built during the feature engineering step was split into training, testing and hold out dataset (Fig 2—step 1). The sample size of the datasets was chosen based on the NORM sample size (training ≈ 70%, testing ≈ 25% and hold out ≈ 5%). Hence, the training dataset contained around 88 trials from each class (≈ 56% of ACL^OP^ and ACL^NO OP^) resulting in a training matrix containing i features and ≈ 264 trials. The testing dataset contained around 30 trials from each class (≈ 20% of ACL^OP^ and ACL^NO OP^) resulting in matrix containing i features and ≈ 90, while the hold out dataset contained the remaining trials. The training dataset was used to teach each machine learning technique to predict the three classes using the previously extracted features. After the training had been completed, class membership of the testing dataset was predicted and compared to the actual class to assess the performance of each learning technique (Fig 2—step 3). When using normalised scores, the testing dataset was normalised using the mean and standard deviation of the training data. The purpose of the hold out dataset was increase the “interchange” of trials within splits, increasing the variation across datasets within the splits and facilitated balanced class distributions within the datasets.

After the stratified shuffle split cross-validation was completed, the learning technique with the highest mean accuracy across the splits was judged most appropriate and used for subsequent analysis (Fig 2—step 5).

Findings during this step demonstrated small differences between classifications accuracies from raw and z-scores (1%). Based on the normalised scores (z-scores), this process identified that the neural network performed best on the DLDJ features (80%), the logistic regression performed best on the SLCMJ (57%), DLCMJ (72%), HuHo (69%) and SLHop (72%) features, while the discriminant analysis performed best on the SLDJ (60%), CoDP (68%) and CoDU (65%) features. The results of this step are described in Table 2 and are visualised in detail in S2 Appendix—Best Learning Technique.

**Table 2:** Description of the performance of the supervised learning techniques using all extracted features within an exercise.

	SLCMJ	DLCMJ	SLDJ	DLDJ	HuHo	SLHop	CoDP	CoPU
Regression	**57**	**72**	59	77	**69**	**72**	67	61
Discriminant	51	59	**60**	60	68	68	**68**	**65**
KnearestNeighbor	42	41	41	42	53	41	41	40
NaiveBayes	54	71	58	76	60	65	59	55
DecisionTree	44	57	46	65	49	59	48	43
RandomForest	55	70	58	80	62	71	60	54
SupportVectorMachine	44	46	45	49	59	54	50	49
NeuralNetwork	53	71	58	**80**	65	68	64	57
Correlation2Mean	36	41	37	42	52	49	40	40
Distance2Mean	39	43	50	38	44	34	39	40

#### Feature selection

**Fig 3:**
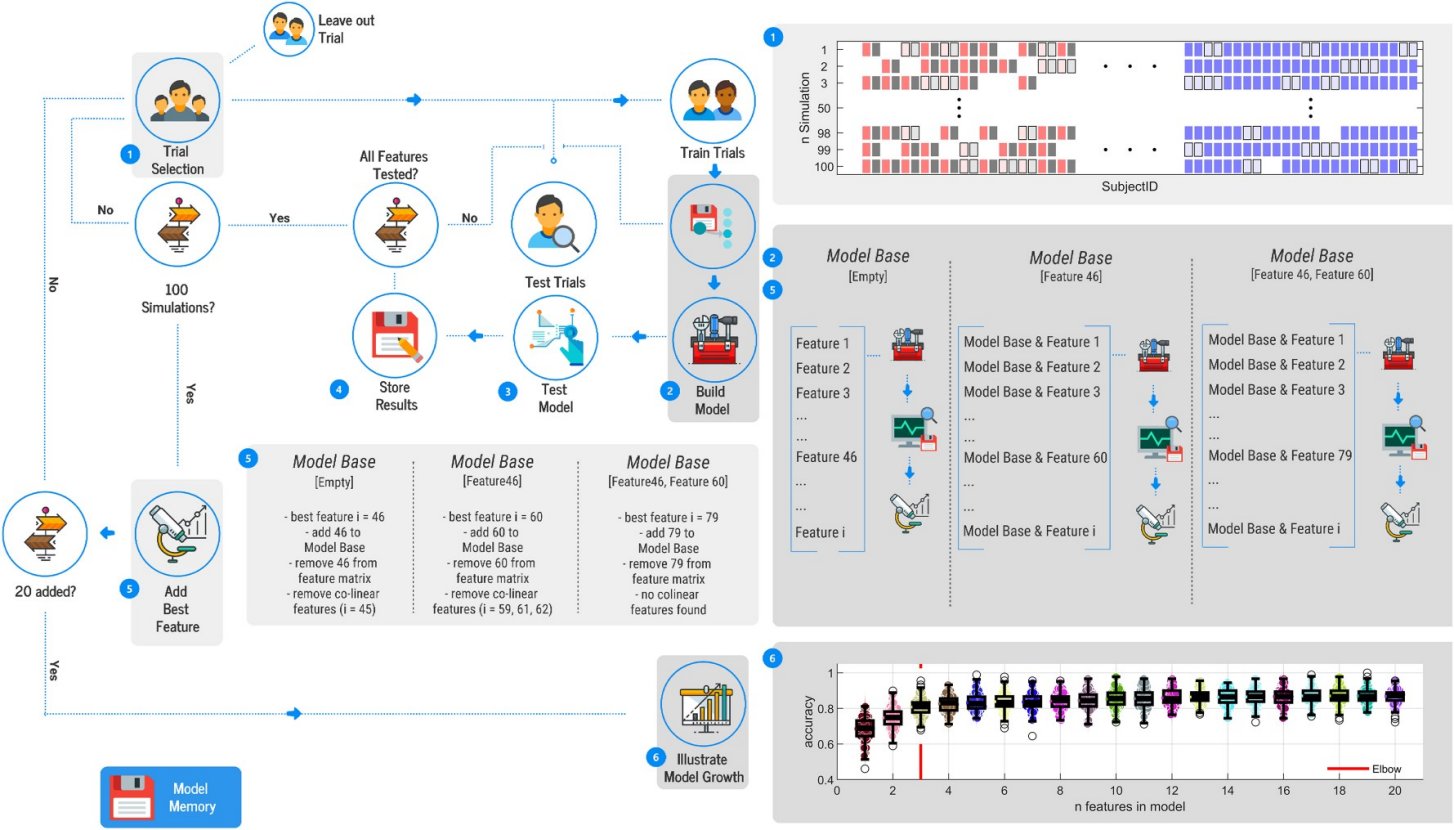
Illustration of the workflow to identify key features toward classification.

The third step was the identification of the features that are most important in the classification. This process used only the best performing machine-learning technique and sought to reduce the effect of over-fitted models by identifying the minimal number of features required to capture most of the information within an exercise. To identify the most important features, a wrapper method (evaluate performance of subsets of features in a forward selection matter) in combination with a stratified shuffle split cross-validation with 100 splits was used (Fig 3). Again, no grid search was performed.

Before starting the selection of important features, an empty matrix (model base) was defined. After the creating of this model base matrix a stratified shuffle split cross-validation with 100 splits was started assessing the classification accuracy of every feature within the feature matrix on its own. In every split the data were divided into training, testing and hold out datasets (Fig 3—step 1). The training dataset was used to teach the previously selected machine-learning technique to predict the three classes (ACL^OP^, ACL^NO OP^ and NORM). After the training phase had been completed, comparing the predicted class of the testing dataset to the actual class assessed the performance of the generated model. After the 100 splits, the feature with the highest mean accuracy was identified, added to the model base matrix and removed from the feature matrix (generated during the feature engineering). All features that correlated with the identified feature (greater than 0.70) were also removed from the feature matrix, to increase interpretability. The value of.70 was a subjective choice as this magnitude is often considered as strong. Subsequently, a second stratified shuffle split cross-validation with 100 splits was started assessing the classification accuracy of the model base matrix in combination with every remaining feature within the feature matrix. The feature that performed best was added to the model base matrix. The just added and any collinear feature where then removed from the feature matrix (Fig 3—step 5). This process was repeated until the model base matrix grew to 20 features (this number was chosen to keep computing time to a reasonable duration; Fig 3—step 6).

The last step during the feature selection was determining how many features should be within a model that presents a good trade-off between efficiency and effectiveness (minimal feature model—e.g. the model with the smallest number of features that accounts for a large part of the contained information). To do this the Elbow method was used—described in Hastie and Tibshirani [45] or Vapnik and Vapnik [46], with the “elbow” being defined as the point n where the differentiation of accuracy f improved less than 10% of its range (Eq 1).

$$\begin{array}{c} f(n+1)-f(n)<[max(f)-min(f)]*.10, \end{array}$$ (1)

The detailed description of results of this step is given in S3 Appendix—Best Features.

#### Final validation of minimal feature model

The number of parameters and models that were tested in this study is large and information could have leaked during the shuffle spilt cross-validation (e.g. possibly over fitting the presented models). To examine if a model was over fitted a 10 fold cross-validation was used to re-evaluate the minimal feature models. During the cross-validation the minimal feature model was trained using 156 subjects (n = 52 per class; ≈90% of Norm and ≈33% of ACL^OP^ and ACL^NO OP^) and validated using 18 subjects (n = 6 per class; ≈9% of Norm and ≈4% of ACL^OP^ and ACL^NO OP^) as testing data. Ten folds were chosen to balance the number of subjects in testing and training with an acceptable accuracy resolution (1 in 18 = 5.56%). The mean accuracy of the 10 fold were then compared to the mean accuracy of the previous used shuffle spilt cross-validation.

Matlab (R2015a, MathWorks Inc., USA) was used for data processing and analysis.

## Results

Findings reported are based on normalised scores (z-scores), for two reasons: the difference between classifications accuracies for the raw and z-scores was small at less than 1% at most and z-scores remove any possible magnitude effect during the selection of features within the optimal model. The highest guess observed during the performed splits was 42%.

### Model performance evaluation

Generated models were generally judged successful in classifying the classes (ACL^OP^, ACL^NO OP^ and NORM). All 3 models generated within an exercise (all features, 20 features, minimal features) were judged meaningful except for the minimal feature for the SLCMJ and CoDU movements. The classification accuracies increased on average 6.1% when reducing the number of features from every extracted feature to 20 features (Table 3). The classification accuracies of the minimal feature models were comparable to the full models (average difference -4.4%; Table 3). When comparing the full model to the minimal feature model: the performance of the DLCMJ, DLDJ, SLHop and HuHo stagnated or improved slightly (0 to 4%), while a slight decrease in performance (-4 to -5%) was observed in the SLCMJ, SLDJ and CoDP. The CoDU minimal feature model decreased its performance by 14% compared to the full model and 15% to the 20 feature model.

**Table 3:** Description of performance observed during the model generation process (using all features, 20 most important features and optimal features.

Exercise	Accuracy best classifier (using all features)	Difference norm vs. raw	Accuracy using only 20 selected features	No. features optimal	Accuracy optimal features	10 fold cross-validation mean ± std	Lowest Accuracy cross-validation
SLCMJ	57%	0%	67%	2	53%	53 ± 10%	40%
DLCMJ	73%	0%	82%	4	73%	62 ± 13%	57%
SLDJ	66%	0%	68%	2	56%	56 ± 11%	45%
DLDJ	82%	0%	87%	3	81%	77 ± 08%	67%
SLHop	75%	1%	79%	5	75%	77 ± 10%	61%
HuHo	67%	1%	74%	5	69%	74 ± 10%	57%
CoDP	66%	1%	77%	5	58%	55 ± 14%	53%
CoDU	66%	0%	67%	2	52%	63 ± 10%	39%

### Minimal feature models: Accuracies, errors and sensitivity

The minimal feature models used between 2 and 5 features (Table 4) and the classification accuracies observed were (in decreasing order): DLDJ (81%), SLHop (74%), DLCMJ (70%), HuHo (69%), CoDP (63%), SLDJ (57%), SLCMJ (53%) and CoDU (52%). The detailed description of results of this step is given in S4 Appendix—Minimal Feature Models.

**Table 4:** Description of minimal feature models: Used machine learning techniques, identified features and corresponding accuracies.

Exercise	Technique	Feature	Accuracy (%)
SLCMJ	neural network model	ankle rotation moment(83-87%)	46
		CoM in pelvis (sagittal plane)(79 to 85%)	53
DLCMJ	logistic regression	knee flexion angle symmetry (46 to 51%)	56
		resultant CoM velocity (87 to 95%)	61
		knee rotation angle symmetry (27 to 33%)	70
		ankle abduction angle symmetry (1 to 13%)	73
SLDJ	discriminant analysis	resultant CoM velocity (94-100%)	51
		vertical CoM velocity (1 to 7%)	56
DLDJ	neural network	vertical ground reaction force symmetry (80 to 86%)	69
		resultant CoM velocity (80 to 84%)	75
		knee flexion angular velocity (74 to 81%)	81
SLHop	logistic regression model	ankle flexion angular velocity (7 to 11%)	58
		CoM in knee(transversal plane) (89 to 100%)	65
		vertical CoM velocity (1 to 7%)	68
		vertical ground reaction force (14 to 19%)	72
		pelvic rotation angles (42 to 50%)	75
HuHo	logistic regression model	knee flexion work (67 to 71%)	48
		vertical ground reaction force (76 to 80%)	56
		knee abduction moment (38 to 42%)	63
		vertical CoM velocity (66 to 76%)	67
		knee flexion angular velocity (84 to 89%)	69
CoDP	discriminant analysis	knee flexion angular velocity (96 to 100%)	48
		knee flexion angle (57 to 61%)	57
		knee abduction moment (52 to 56%)	63
CoDU	discriminant analysis	vertical CoM velocity (86 to 91%)	48
		hip flexion moment (91 to 95%)	52

Results for the 10-fold cross-validation of the minimal feature model demonstrated differences in mean accuracy to the shuffle-split cross-validation (Table 4; in decreasing order): DLCMJ (-11%), CoDU (+11%), SLHop (5%), DLDJ (-4%), CoDP (-3%), HuHo (+3%), SLDJ (0%) and SLCMJ (0%).

Classification errors originated from misclassifications of the class ACL^OP^ and ACL^NO OP^ in the optimal SLHop and HuHo model (Table 5). In these models the percentage of misclassification within the ACL group was higher (≈ 2 times) than misclassification of either ACL class with NORM, while the SLCMJ model tended to misclassify both ACL classes with NORM class. The DLCMJ and DLDJ results displayed increased misclassifications between NORM and either ACL class compared to between the ACL classes (≈ 1.8 and 4 times; Table 5). The SLDJ displayed increased misclassifications (≈ 2 times) between the ACL^NO OP^ and NORM class compared to ACL^OP^ with ACL^NO OP^ or NORM (Table 5). In the CoDP and CoDU models, misclassifications were equally distributed between the classes (Table 5).

**Table 5:** Description of the confusion matrix for the minimal feature model of every examined exercise: Reporting true positive, true negative, false positive and false negative as count (percentage across all classes).

SLCMJ	ACL^OP^	ACL^NO OP^	NORM	DLCMJ	ACL^OP^	ACL^NO OP^	NORM
ACL^OP^	1928 (22%)	526 (6%)	514 (6%)	ACL^OP^	2190 (24%)	339 (4%)	471 (5%)
ACL^NO OP^	825 (9%)	1562 (18%)	525 (6%)	ACL^NO OP^	338 (4%)	2190 (24%)	472 (5%)
NORM	909 (10%)	901 (10%)	1092 (12%)	NORM	512 (6%)	504 (6%)	1959 (22%)
SLDJ	ACL^OP^	ACL^NO OP^	NORM	DLDJ	ACL^OP^	ACL^NO OP^	NORM
ACL^OP^	2012 (28%)	421 (5%)	542 (6%)	ACL^OP^	2501 (28%)	108 (1%)	391 (4%)
ACL^NO OP^	604 (7%)	1463 (17%)	908 (10%)	ACL^NO OP^	109 (1%)	2507 (28%)	384 (4%)
NORM	525 (6%)	902 (10%)	1460 (17%)	NORM	355 (4%)	334 (4%)	2282 (25%)
SLHop	ACL^OP^	ACL^NO OP^	NORM	HuHo	ACL^OP^	ACL^NO OP^	NORM
ACL^OP^	2088 (23%)	657 (7%)	255 (3%)	ACL^OP^	2007 (22%)	658 (7%)	321 (4%)
ACL^NO OP^	587 (3%)	2129 (24%)	284 (3%)	ACL^NO OP^	657 (7%)	1803 (20%)	523 (6%)
NORM	267 (3%)	291 (3%)	2370 (27%)	NORM	175 (2%)	389 (4%)	2388 (27%)
CODP	ACL^OP^	ACL^NO OP^	NORM	CoPU	ACL^OP^	ACL^NO OP^	NORM
ACL^OP^	1828 (21%)	566 (6%)	558 (6%)	ACL^OP^	1705 (20%)	659 (8%)	554 (7%)
ACL^NO OP^	706 (8%)	1640 (19%)	552 (6%)	ACL^NO OP^	795 (9%)	1326 (16%)	717 (8%)
NORM	364 (4%)	485 (6%)	2030 (23%)	NORM	567 (7%)	784 (9%)	1410 (17%)

When assessing the performance of minimal feature models on a group level (ACL [ACL^OP^ and ACL^NO OP^] and NORM) the single leg exercises demonstrate large improvements (12 to 24%), while the double leg exercises did not (2 to 8%; Table 6).

**Table 6:** Description of class specific sensitivity, performance of binary classification (ACL and NORM) and percentage of the athletes within the NORM group that were never confused.

Exercise	% for Norm class	% for ACL^OP^ class	% for ACL^NO OP^ class	% binary classifier (delta to multi class)	% of sample representing true Norm
SLCMJ	51	53	52	67 (+15)	27
DLCMJ	68	72	72	78 (+8)	43
SLDJ	50	64	53	69 (+12)	39
DLDJ	75	84	85	83 (+2)	52
SLHop	81	71	69	88 (+24)	71
HuHo	74	71	63	83 (+12)	70
CoDP	65	63	61	77 (+14)	65
CoDU	53	56	48	70 (+17)	40

The sensitivity for the classification of the NORM class was highest in the SLHop (81%), while for the sensitivity for ACL^OP^ and ACL^NO OP^ trials was highest for the DLDJ (84 and 85%; see Table 6).

The percentage of the athletes within the NORM group that were never confused (’true’ NORMs) ranged from 27 to 71% depending on the exercise (Table 6). The percentage of the athletes within the ACL^NO OP^ and ACL^OP^ group that were never confused ranged from 52 to 85% depending on the exercise (Table 6).

## Discussion

This study examined the classification accuracy of a framework that combines a data-driven feature extraction procedure that did not require expert knowledge and a supervised machine learning technique to differentiate movement patterns from ACL operated class (ACL^OP^), from a limb contralateral to an ACL operated limb (ACL^NO OP^) and a non-injured control limbs (NORM) using only biomechanical data. Findings demonstrate that biomechanical data, regardless of the exercise, contained sufficient information to outperform guessing and suggest that it is possible to differentiate normal movement patterns from movement patterns after an ACL reconstruction on a group and limb level.

### Model evaluation

#### Classification accuracy

This study developed a minimal feature model in 3 steps that generated three prediction models: one that utilised a large number of features, one model utilised only 20 (most important) features and a minimal feature model. The minimal feature model contained 2 to 5 features and classification accuracies were similar between the three generated models. For the DLCMJ, DLDJ, HuHo and SLHop, using the minimal feature models reduced the information utilised by 94 to 98% (160 features vs. ≈ 4 features) but achieved performances similar (-2 to 2%) to models using all generated features. In contrast, the performance of the minimal feature model decreased for the SLCMJ, SLDJ, CoDP and CoDU in comparisons to the model using all generated features (-5 to -12%). The decrease in accuracy in the minimal feature model SLCMJ, SLDJ, CoDP and CoDU indicates an increased complexity in these exercises, compared to the DLCMJ, DLDJ, HuHo and SLHop, and suggests that these models need more information (features) to perform as well as they could. However, all exercises improved performances when reducing the number of features from 97 to 186 to only 20, which is a reduction of used features of ≈ 84% (138 features vs. 20 features), by ≈ 7%. This suggests that there was an effect of over-fitting to the training data in the models that used all extracted features and highlights that the importance of some kind of feature selection before model building using biomechanical analysis using comparable sample sizes. However, this finding also highlights that a relatively small number of biomechanical features (between 2 and 20) captures a large amount of the information content from a biomechanical assessment. Regarding the information contained within the examined movement data when assessing classification accuracies in a multi-class classifier, classification models achieved accuracies of (in decreasing order): DLDJ (81%), SLHop (75%), DLCMJ (73%), HuHo (69%), CoDP (58%), SLDJ (56%), SLCMJ (53%) and CoDU (52%). Little research exists currently that used biomechanical data to classify group / cohort membership. The closest research is that of Ross et al., [10] who analysed the movements in elite and non-elite performers and found that a data driven model could classify an individual into novice and elite with an classification accuracies between 71 to 80% across 13 exercises. While the classification accuracies of the SLCMJ, SLDJ, CoDP and CoDU seem to be low in comparison to other exercises or the findings of Ross et al., [10], it should be considered that their performance was assessed in an multi-class classifier. The classification accuracy of the SLCMJ, SLDJ, CoDP and CoDU in a binary or group classifier (ACL or NORM) was 67, 69, 77 and 70%, making them comparable to reported classification accuracies observed by Ross et al., [10].

#### Classification errors

Classification errors in the tested multi-class classifiers could have occurred due to a reduced / smaller difference in movement within the ACL classes compared to the ACL and NORM classes—e.g. because of anatomical / morphological differences between the groups (ACL and NORM). When assessing the performances on a group level (ACL and NORM) performances improved significantly, compared to the limb class level, for every single leg exercise (≈ 16%) but not for double leg exercises (≈ 5%), suggesting differences between the single and double leg exercises. These differences might be explained by the additional symmetry information that was contained in the double exercises but not in the single leg exercises. This assumption is supported by the fact that in the double leg exercises, symmetry features were selected frequently (4 out of 7). The magnitude of symmetry or asymmetry seems to be a useful feature within the generated models, which supports findings of Myer et al., [47] and others [48–50]. While symmetry features could have been included within the single leg models, it requires the intervention of the investigators—e.g. symmetry calculation as mean symmetry, symmetry between trial x and trial y and so on [51]. Symmetry was not included in this study because it cannot be calculated without setting subjective rules (e.g. expert knowledge), as every execution presents different external and internal conditions. Nevertheless, findings suggest that symmetry measures are important and hence they should have been considered.

**Fig 4:**
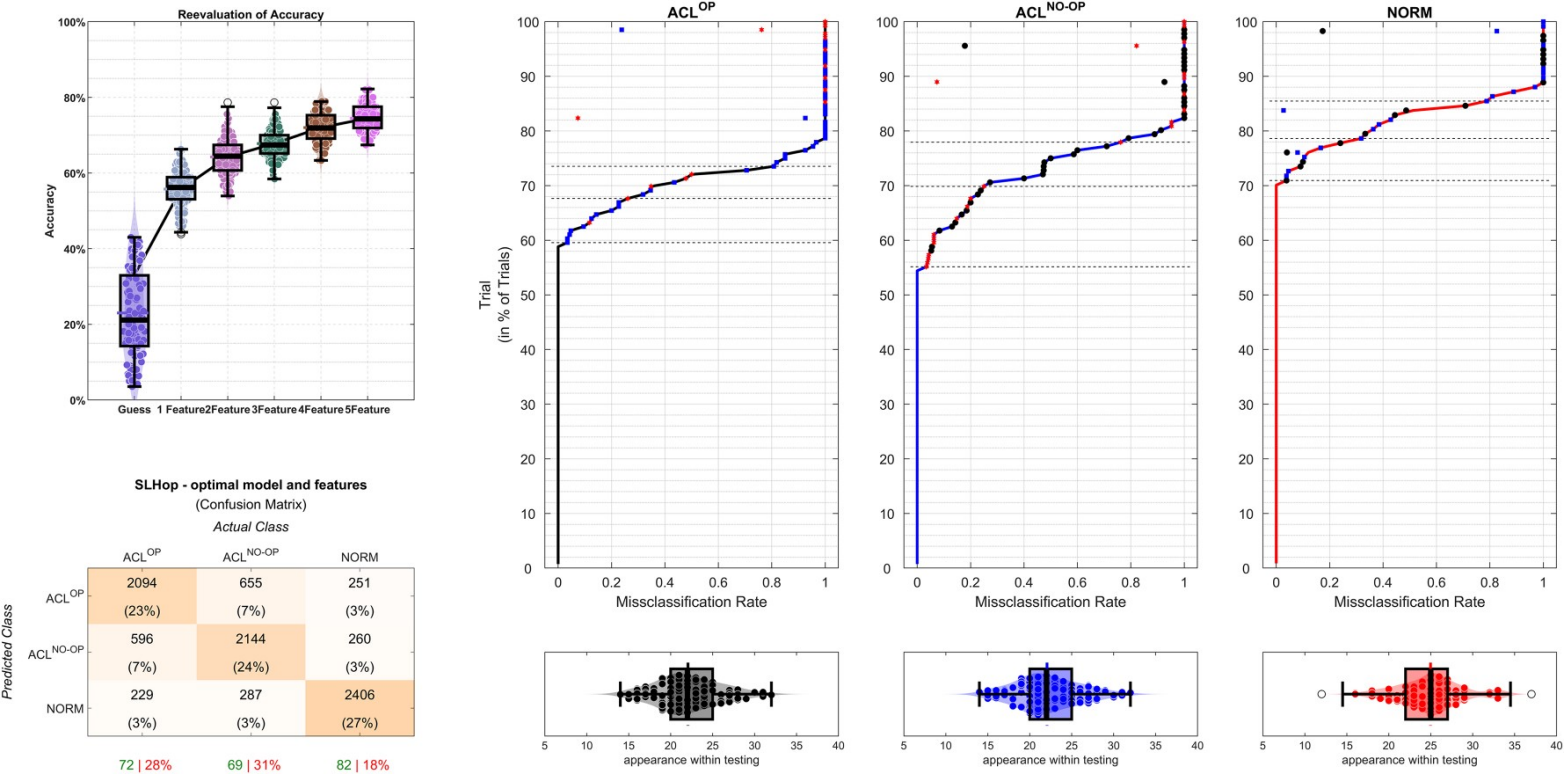
Illustration of the results of the SLHop model: The model accuracy is displayed on the top right, while the confusion matrix of a 2-feature model is displayed below (right button). The three graphs on the left display the confusion pattern (button) and selection frequency of each trail within the ACL^OP^, ACL^NO OP^ and NORM (top).

Regarding errors that might have been caused by anatomical or morphological differences between the groups, a large part of error in the SLHop and HuHo classification errors originated from confusing ACL^OP^ and ACL^NO OP^ (see Fig 4). In these models the percentage of misclassification within the ACL group was higher (≈ 2 times) than confusing either ACL class with NORM. This suggests that the movement of both limbs in the ACL group is affected by an ACL reconstruction—as both limbs can be differentiated from the NORM pattern but less from each other. As such, the ACL^NO OP^ may not be ideal reference when judging the rehabilitation status and readiness to return to play. Another reason could be that the differences between ACL^OP^ and ACL^NO OP^ are not well described in the examined features.

**Fig 5:**
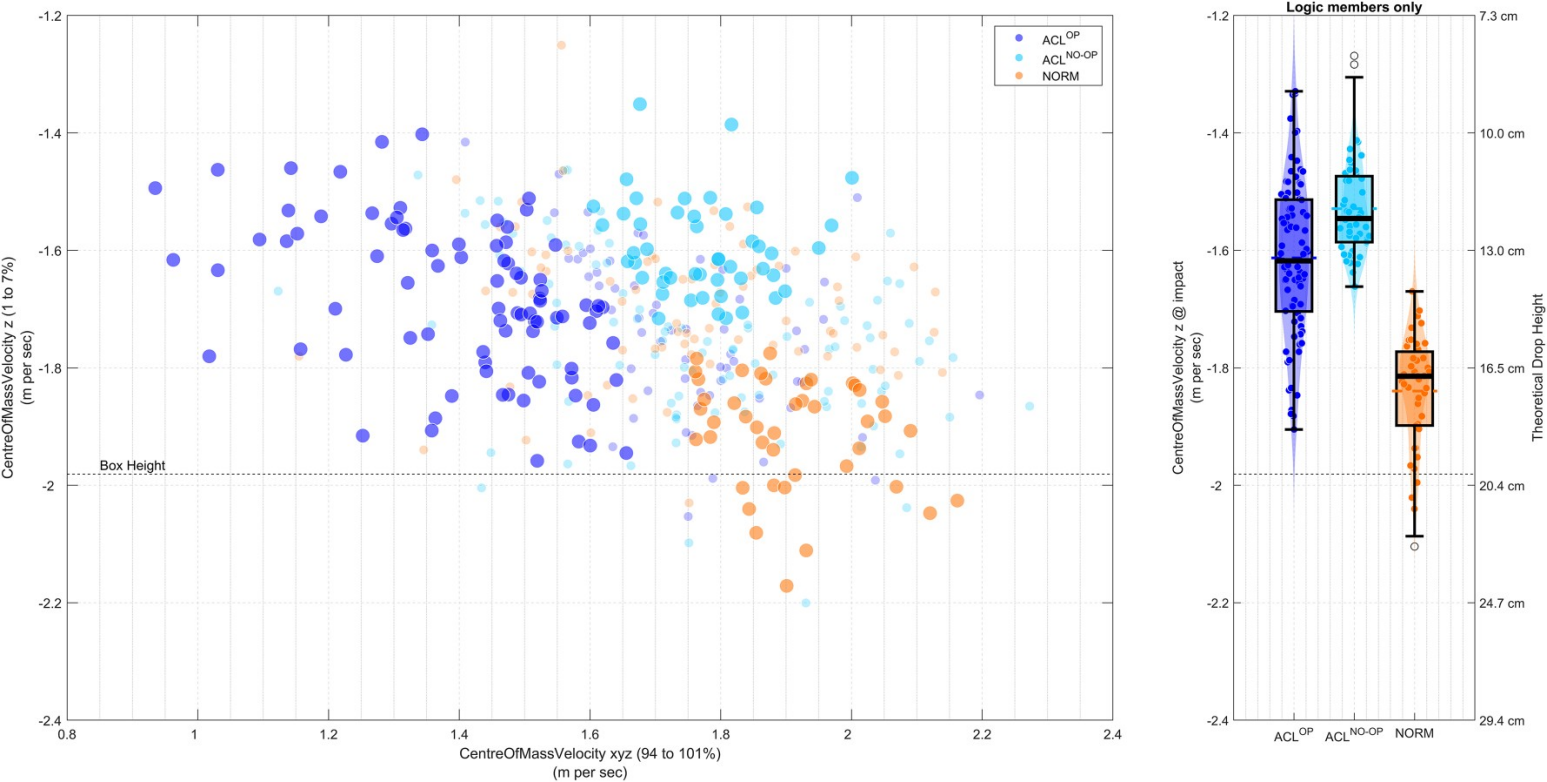
Illustration of the 2 most important SLDJ model features (CoM resultant velocity at take off values and vertical CoM velocity at impact) plotted against each other on the left and the CoM velocity at impact for the logic cases on the right. Fully coloured bubbles represent trials with ‘true’ group pattern, while faded coloured bubbles represent trails that did not.

The SLDJ, DLDJ, SLCMJ, CoDP and CoDU models demonstrated different error or misclassification patterns. The SLDJ would have benefited most from a binary-classifier that classified for ACL reconstructed limb (ACL^OP^) and not ACL reconstructed limb (ACL^NO OP^ and NORM), as a large part of misclassifications were made by confusing ACL^NO OP^ and NORM (confusions within the ACL group were less common). This would suggest that the SLDJ is able to detect differences between a limb with ACL reconstruction and limb without ACL reconstruction—implying that the ACL^NO OP^ limb could be used as reference when judging the progress of rehabilitation. The interpretation of the selected features in the minimal feature model, however, suggests that the execution of the exercise was modified for within the ACL classes and between the groups. The vertical CoM velocity at impact was selected yet it should not hold any meaningful information, as it should be nearly the same (impact velocity should theoretically be 1.981 m/sec.) across all trails because of the controlled drop height. Inspection the model visually reveals: a) that the ACL^NO OP^ and NORM class demonstrate similar CoM resultant velocity at take off values, while values in ACL^OP^ are reduced, and b) that the vertical CoM velocity at impact values are different between ACL^NO OP^ and NORM and spanned over the range of classes for the ACL^OP^ class (Fig 5). Based on a post hoc analysis, the ACL^NO OP^ and some of the ACL^OP^ class have ‘changed’ the drop height by adapting a stepping down pattern (Fig 5), which was not detected and resolved during the biomechanical assessment, in spite of careful instruction and frequent lab quality assessments. This highlights is the importance of interrogating what the model has learned during a data driven process as other features should be been normalised to the ‘changed’ the drop height and highlights possible psychological differences between the group [52].

In the DLCMJ and DLDJ, errors originated from misclassifying NORM with either ACL class or both ACL classes with NORM, with the DLDJ demonstrating a larger inequality in distribution (≈ 1.8 vs. 4 times). This might be explained by an increase of the physical demand of the DLDJ that poses an additional challenge to symmetry and reactivity. While the minimal feature SLCMJ model demonstrated classification errors that originated equally from confusion between the classes, the 20 feature or all feature SLCMJ model displayed that confusion between ACL^NO OP^ and NORM were ≈ 2 times higher compared to other classification errors. This suggests that models that utilised more features possibly detected some patterns that differ between ACL operated and not operated limbs (when being provided with information from many features). Similar to the SLCMJ the minimal feature CoDP and CoDU models demonstrated classification errors that originated equally from confusion between classes, while the 20-feature model displayed an increased error from misclassification within the ACL group (≈ 2 times). This would suggest that the ACL^OP^ and ACL^NO OP^ class share more similarity with each other than to the NORM class.

#### Classification sensitivities

Another source of error for the models is the misclassification of specific athletes within a class that does not present the ‘true’ class movement patterns. When examining the prediction errors on an athlete level, it was noted that some athletes are always classified correctly; some are occasionally classified correct, while some are always misclassified. This was true for all classes and exercises. The detection of members within a group that are not always classified correctly could help to understand group differences [30] as they could be excluded from subsequent analysis (as done in Fig 5 to better understand the class pattern the model has learned). The existence of such athletes or the proportion of such athletes within a group may also explain why there are so many conflicting findings in inference based studies. However, knowing that such athletes exist also enables the development of our understanding of injuries as the probability of belonging to a desired class computed during a classification could be used when judging movement analysis and injury risk. Consequently, the probability of membership to a class (in this case the NORM class) could also allow the objective judgment of a movement or the rehabilitation progress. The probability of membership can give an objective measure of how close a trial is to a desired class and presents a clear criterion if an athlete has returned to normal.

### Practical implications

Return to play after ACL reconstruction as well as the prevention of subsequent re-injury is not always guaranteed [53, 54] and this might be in part due to absence of clear criteria identifying if an athlete has returned to pre-injury levels or completed rehabilitation. Current clinical testing batteries often utilise biomechanics to assess a movement quality. However, there is little consensus on the appropriateness of biomechanical analysis and / or specific exercise tests and measures when differentiating between two specific groups. This study demonstrates that biomechanical data holds enough information to differentiate between ACL^OP^, ACL^NO OP^ and NORM with classification accuracies above 70%, even if the ACL group was 9.4 ± 0.7 months post-op and most had completed their rehabilitation.

Findings suggest that classification models from different exercises capture different pieces of information and hence a variety of exercises should be used. A biomechanical assessment should contain measures of symmetry and chosen exercises might be altered throughout the rehabilitation process to increase complexity, while familiarising the athlete to the demands of sports. The selection of a single exercise test cannot be recommended as all examined exercises demonstrated that they contain valuable information and we did not adjust parameters within the models that could lead to improvements in classification accuracy.

Findings highlight problems with the assumption that the majority of a control group demonstrate healthy movement patterns, which could be a reason for conflicting findings in studies. Within the examined exercises only 27 to 71% of the limbs within NORM did present a true NORM pattern.

### Limitations

#### Examined data

Like most movement analysis studies, this study involved the recording of multiple trials but examined only the best trial. Examining the best trial is likely to be more valid than averaging multiple trials, which creates and examines an artificial movement where local peaks are altered in magnitude and temporal appearance [55] and the intersegmental link (coordination) between joints might be lost. However, the approach used selects a unique instance (maximal performance) and could bias an analysis towards a non-realistic situation. No athlete will perform a task over and over with a maximal effort and consequently the sub-maximal efforts should not be discarded. An alternative approach is to utilise repeated random sampling, where the captured trials are selected at random and the analysis is run multiple times [56–58]. This can overcome the ‘maximal effort bias’ and can also provide a measure of expected differences or accuracy within future studies. Further, such an approach can also overcome discrepancies between findings that are caused by the selection of a reference limb when comparing an abnormal (injured) to a normal or uninjured group.

#### Examined features

The use of waveform analysis and total exclusion of discrete points ignores the magnitudes and location of peak values that might be important. For example, angular velocity and GRF time series can be multimodal (multiple local maxima’s) and maximal or minimal might not be located in the same phases and the information of these features is hence lost. Such features and their temporal properties (position) might carry important information that has been discarded in this study. Further, the manner in which the data driven framework approached the engineering feature was based on no input of expert knowledge (e.g. complexity [59], stiffness [60], variability [61]) or any advanced feature engineering techniques (principal component scores, non negative matrix factorisation or interactions between features). No additional engineering feature was used to keep the interpretable and meaningful for rehabilitation, while additional more complex features are likely to increase the reported classification accuracies.

#### Model building

This study tested machine learning techniques using only their default values and did not perform a grid search (optimise adjustable parameters within a technique; e.g. changing L1 and L2 regularisation in logistic regression or changing k in the k-nearest neighbour technique), which would very likely have resulted in higher classification accuracies and might impact the selected features in the optimal model. Additionally, for the DLCMJ and CoDU findings should be interpreted with care as findings from a 10-fold cross-validation possibly indicate over-fitting for the DLCMJ model and an under-fitting for the CoDU model.

## Conclusion

This study tested a data-driven framework that required no expert or prior knowledge and demonstrated that biomechanical data can predict with high accuracy (≈ 70%) if a movement was performed by a limb following ACL reconstruction, the contralateral limb and a limb of a healthy control group. Findings highlight that a few features can contain most of the information content, that symmetry measures are important, that it is important to seek to understand what a classification / prediction model has learned, that different exercises capture different movement characteristics and that not all subjects within a normative cohort utilise a ‘true’ normative movement pattern.

## Supporting information

S1 Appendix Detected phases. (PDF)

S2 Appendix Best learning technique. (PDF)

S3 Appendix Best features. (PDF)

S4 Appendix Minimal feature models. (PDF)
