# Supplementary material for: Objective classification and scoring of movement deficiencies in patients with anterior cruciate ligament reconstruction
Source: PLoS One. 2019 Jul 23;14(7):e0206024. doi: 10.1371/journal.pone.0206024 (PMC6650047; doi:10.1371/journal.pone.0206024)
Supplement: S1 Appendix — (PDF) [file pone.0206024.s001.pdf]

Appendix S1 - Detected phases of variation

This appendix reports findings of the phase detection step within each exercise.

During the feature generation process subjects from the ACL group have been included on average 27 times (min 0 - max 38) into the data set, while subject within the NORM class were included on average 68 times (min 58 - max 78). The generated feature matrix for the SLCMJ contained 140 features (fig 1).

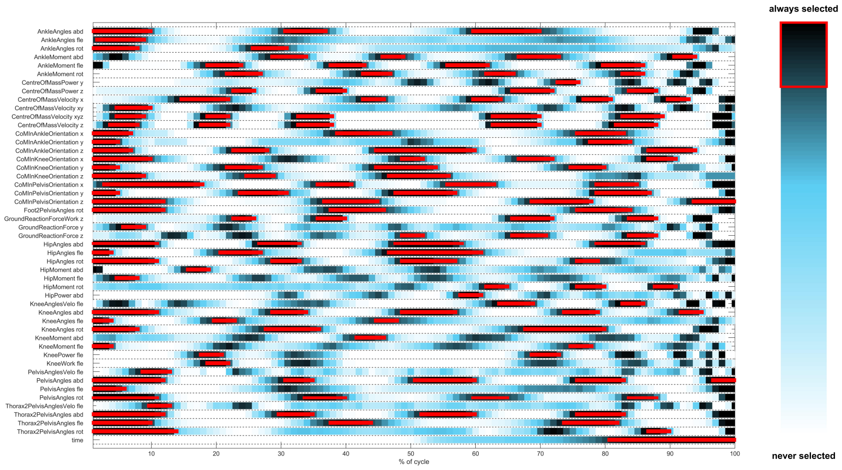

Figure 1: Illustration of detected phases of variation within the SLCMJ. The color intensity represents the frequency of a frame to be identified as phases of variation, while the red bars highlights phases that meet the selection criteria and were included within the feature matrix.

The generated feature matrix for the DLCMJ contained 176 features (fig 2).

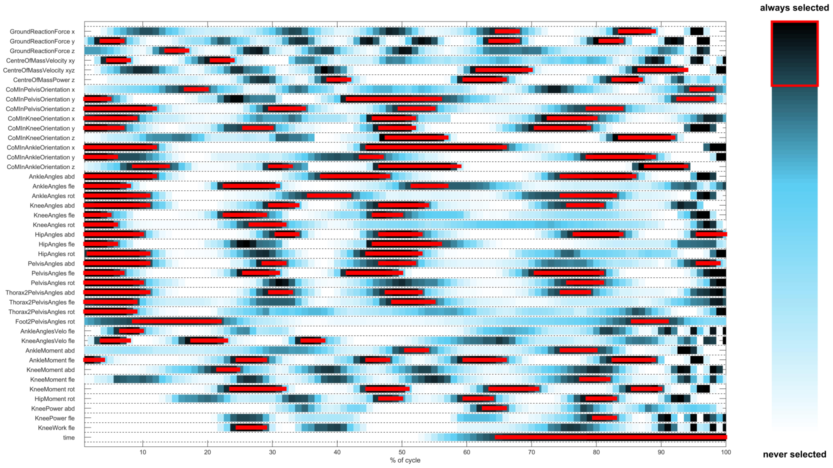

Figure 2: Illustration of detected phases of variation within the DLCMJ. The color intensity represents the frequency of a frame to be identified as phases of variation, while the red bars highlights phases that meet the selection criteria and were included within the feature matrix.

The generated feature matrix for the SLDJ contained 97 features (fig 3).

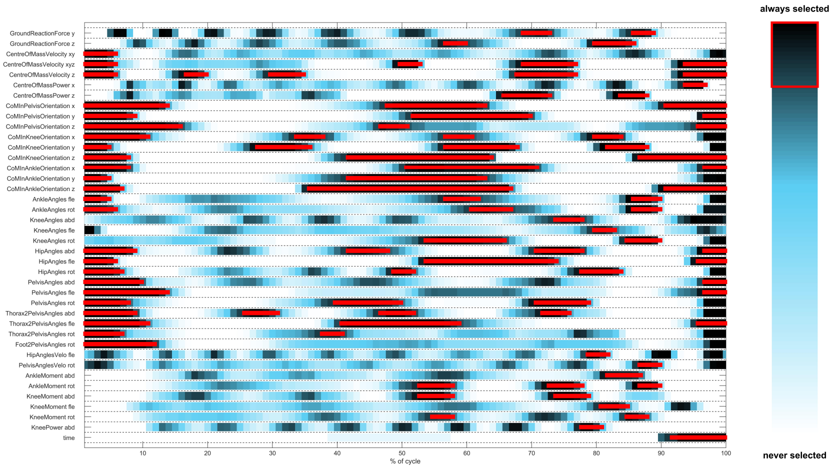

Figure 3: Illustration of detected phases of variation within the SLDJ. The color intensity represents the frequency of a frame to be identified as phases of variation, while the red bars highlights phases that meet the selection criteria and were included within the feature matrix.

The generated feature matrix for the DLDJ contained 186 features (fig 4).

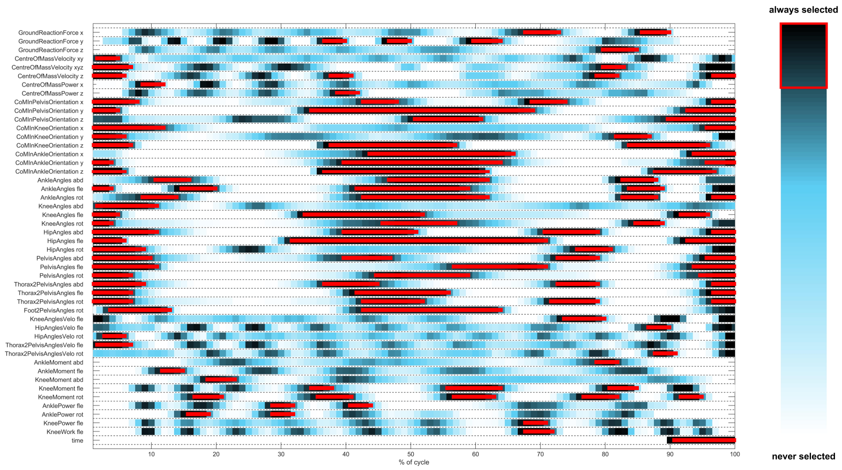

Figure 4: Illustration of detected phases of variation within the DLDJ. The color intensity represents the frequency of a frame to be identified as phases of variation, while the red bars highlights phases that meet the selection criteria and were included within the feature matrix.

The generated feature matrix for the HuHo contained 119 features (fig 5).

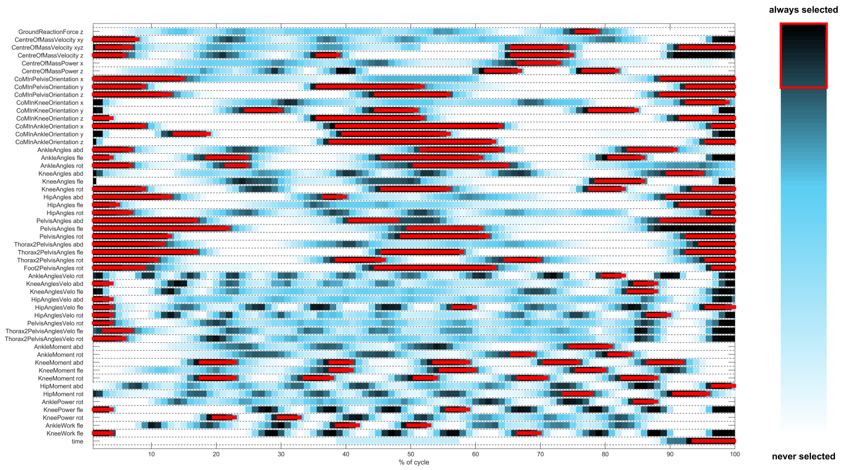

Figure 5: Illustration of detected phases of variation within the HuHo. The color intensity represents the frequency of a frame to be identified as phases of variation, while the red bars highlights phases that meet the selection criteria and were included within the feature matrix.

The generated feature matrix for the SLHop contained 157 features (fig 6).

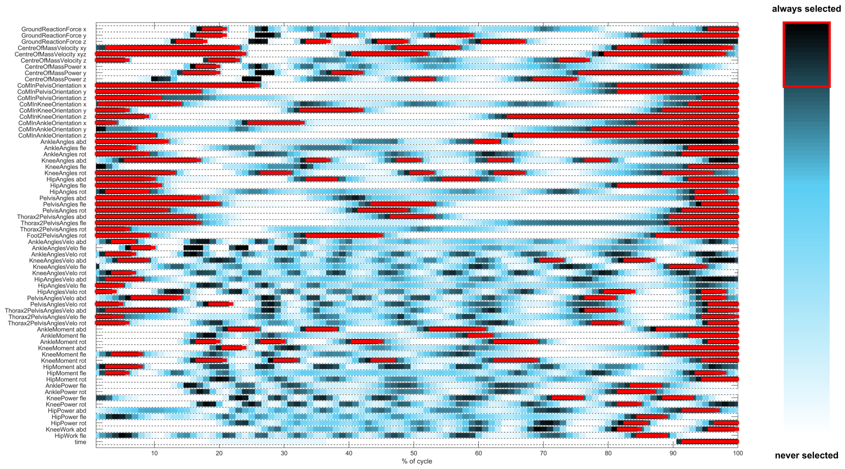

Figure 6: Illustration of detected phases of variation within the SLHop. The color intensity represents the frequency of a frame to be identified as phases of variation, while the red bars highlights phases that meet the selection criteria and were included within the feature matrix.

The generated feature matrix for the CoDP contained 132 features (fig 7).

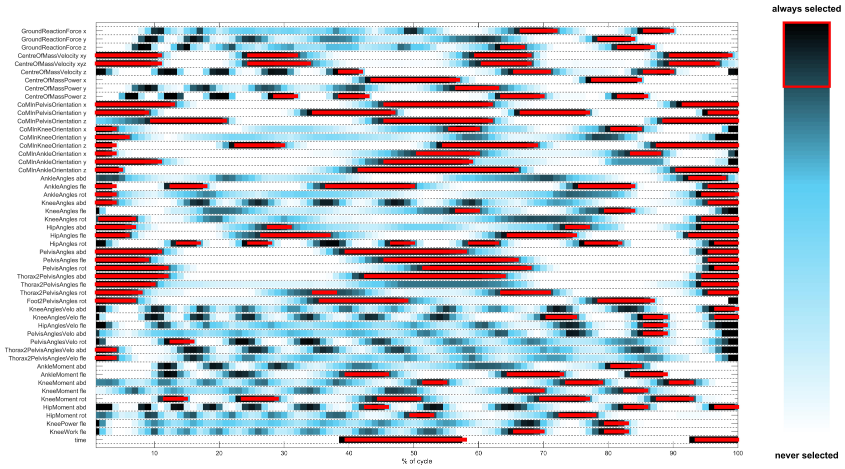

Figure 7: Illustration of detected phases of variation within the CoDP. The color intensity represents the frequency of a frame to be identified as phases of variation, while the red bars highlights phases that meet the selection criteria and were included within the feature matrix.

The generated feature matrix for the CoDU contained 97 features (fig 8).

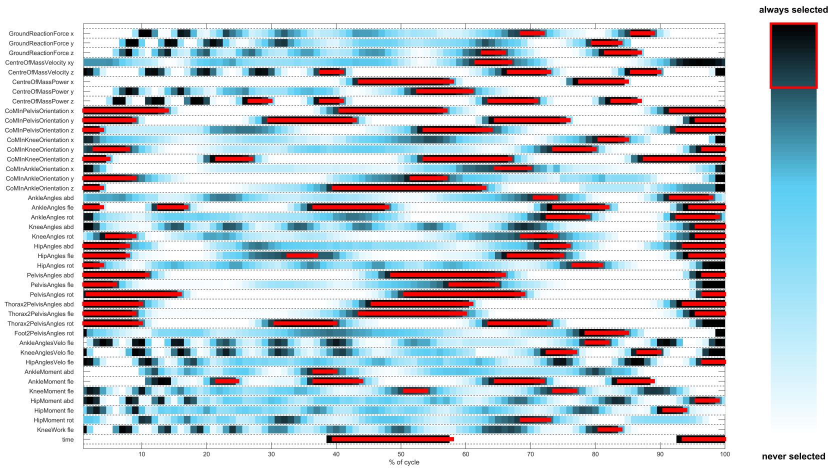

Figure 8: Illustration of detected phases of variation within the CoDU. The color intensity represents the frequency of a frame to be identified as phases of variation, while the red bars highlights phases that meet the selection criteria and were included within the feature matrix.
