# Supplementary material for: Objective classification and scoring of movement deficiencies in patients with anterior cruciate ligament reconstruction
Source: PLoS One. 2019 Jul 23;14(7):e0206024. doi: 10.1371/journal.pone.0206024 (PMC6650047; doi:10.1371/journal.pone.0206024)
Supplement: S2 Appendix — (PDF) [file pone.0206024.s002.pdf]

Appendix S2 - Machine Learning Technique Selection

This appendix reports findings of the machine learning selection step within each exercise.

During the this process subjects from the ACL group have been included on average 60 times (min 49 - max 73) into the trainings data set and on average 18 times (min 8 - max 32) into the test data set. Subjects of the NORM group were included on average 75 times (min 65 - max 83) into the trainings data set and on average 22 times (min 13 - max 33) into the test data set. While normalising features did not effect the magnitude of best the classification accuracy (differences around 1 %), it did effect the ranking order of the learning techniques. As the performance was not impacted, findings reported and subsequent model generation was done using normalised features - because z-scores remove any possible magnitude effect during the selection of features for the final model.

The neural network performed best when examining the DLDJ (80 $\pm$ 5 %; fig 4). The logistic regression performed best when examining the SLCMJ (57 $\pm$ 5 %; fig 1), DLCMJ (72 $\pm$ 6 %; fig 2) and SLHop (72 $\pm$ 4 %; fig 6), while the discriminant analysis performed best features from the SLDJ (60 $\pm$ 5 %; fig 3), CoDP (68 $\pm$ 5 %; fig 7) and CoDU (65 $\pm$ 5 %; fig 8).

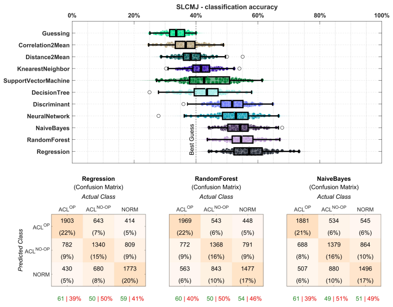

(a) Raw scores.

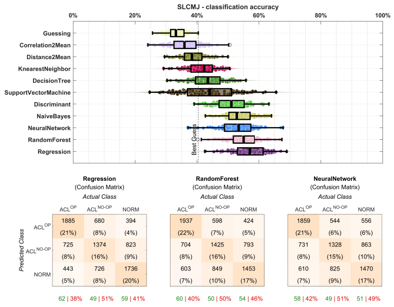

(b) Normalised Scores.

Figure 1: Illustration of the achieved performance of the tested techniques within the SLCMJ.

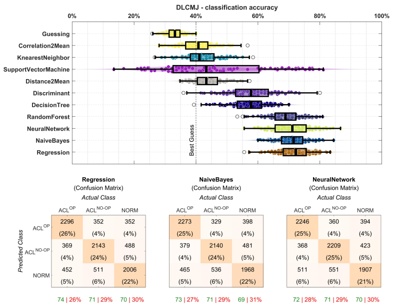

(a) Raw scores.

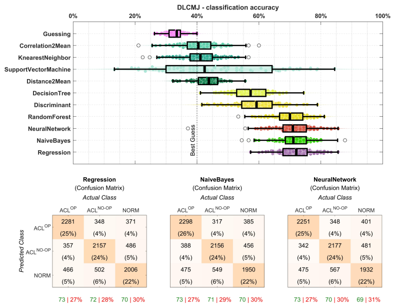

(b) Normalised Scores.

Figure 2: Illustration of the achieved performance of the tested techniques within the DLCMJ.

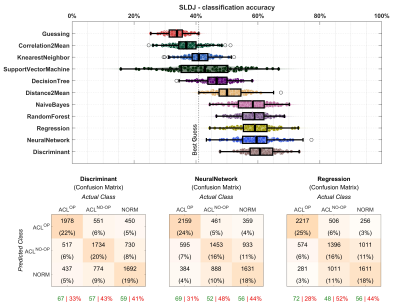

(a) Raw scores.

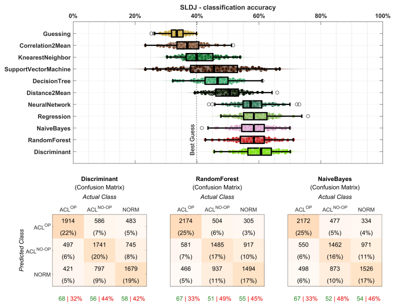

(b) Normalised Scores.

Figure 3: Illustration of the achieved performance of the tested techniques within the SLDJ.

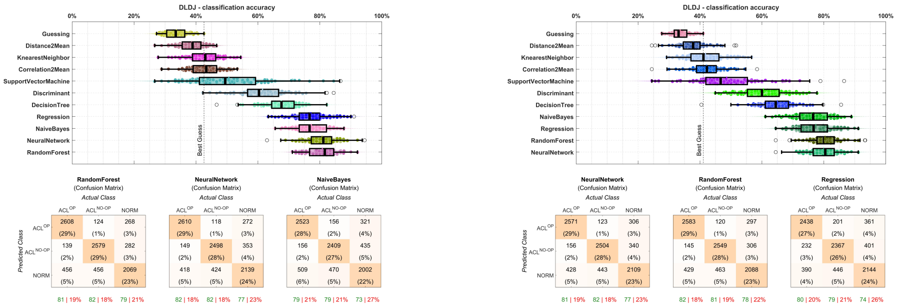

(a) Raw scores.

(b) Normalised Scores.

Figure 4: Illustration of the achieved performance of the tested techniques within the DLDJ.

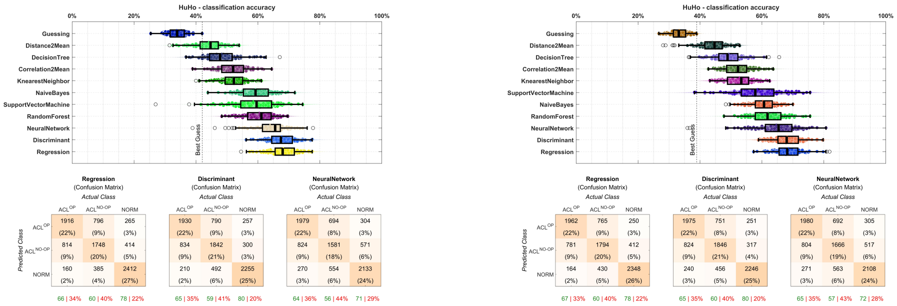

(a) Raw scores.

(b) Normalised Scores.

Figure 5: Illustration of the achieved performance of the tested techniques within the HuHo.

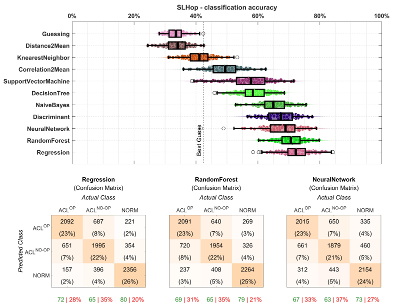

(a) Raw scores.

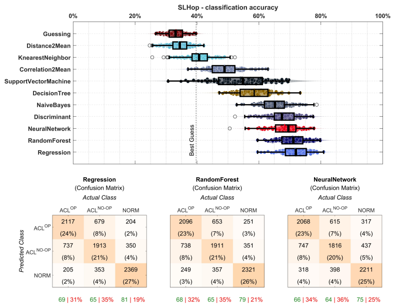

(b) Normalised Scores.

Figure 6: Illustration of the achieved performance of the tested techniques within the SLHop.

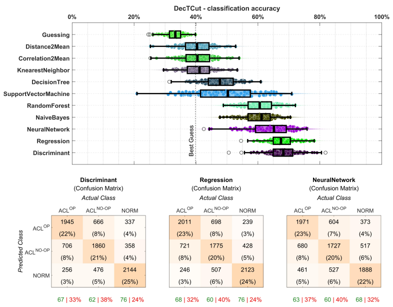

(a) Raw scores.

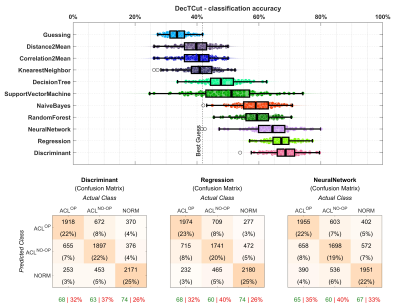

(b) Normalised Scores.

Figure 7: Illustration of the achieved performance of the tested techniques within the CoDP.

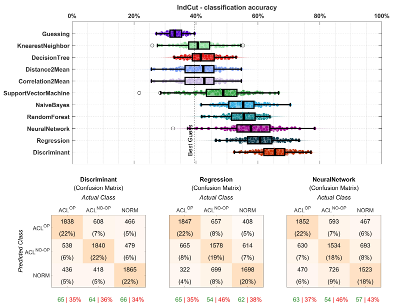

(a) Raw scores.

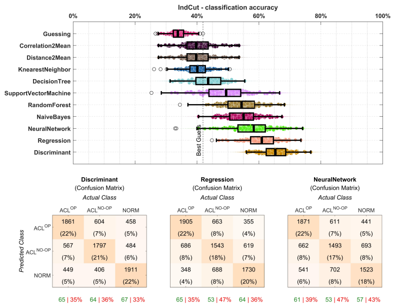

(b) Normalised Scores.

Figure 8: Illustration of the achieved performance of the tested techniques within the CoDU.
