# Supplementary material for: Objective classification and scoring of movement deficiencies in patients with anterior cruciate ligament reconstruction
Source: PLoS One. 2019 Jul 23;14(7):e0206024. doi: 10.1371/journal.pone.0206024 (PMC6650047; doi:10.1371/journal.pone.0206024)
Supplement: S3 Appendix — (PDF) [file pone.0206024.s003.pdf]

Appendix S3 - Feature Selection

This appendix reports findings of the feature selection step within each exercise.

During the process subjects from the ACL group have been included on average 60 times (min 49 - max 73) into the training data set and on average 18 times (min 8 - max 32) into the test data set. Subjects of the NORM group were included on average 75 times (min 65 - max 83) into the training data set and on average 22 times (min 13 - max 33) into the testing data set.

Selected features in the SLCMJ model were: ankle rotation moment$^{(83\text{-}87\ \%)}$ and CoM in pelvis (sagittal plane)$^{(79\text{ to }85\ \%)}$. The neural network model with this two features achieved an average accuracy of 53 %. A detailed illustration of the findings is displayed in figure 1.

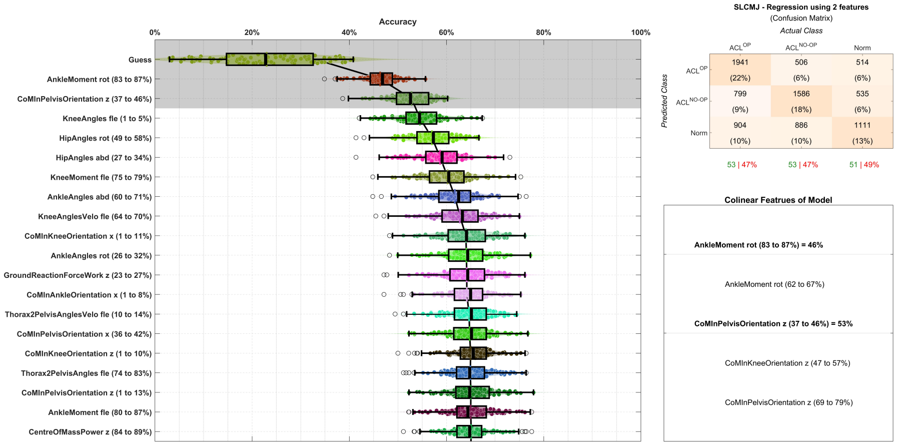

Figure 1: Illustration of the SLCMJ results. The left side displays the accuracy of the model and the utilised features. The top right displays the confusion matrix of the model using the grey shaded features. Button right reports the selected features and co-linear features.

Selected features in the DLCMJ model were: knee flexion angle symmetry $^{(46\text{ to }51\ \%)}$, resultant CoM velocity $^{(87\text{ to }95\ \%)}$, knee rotation angle symmetry $^{(27\text{ to }33\ \%)}$ and ankle abduction angle symmetry $^{(1\text{ to }13\ \%)}$. The logistic regres-

sion model with this four features achieved an average accuracy of 73 %. A detailed illustration of the findings is displayed in figure 2.

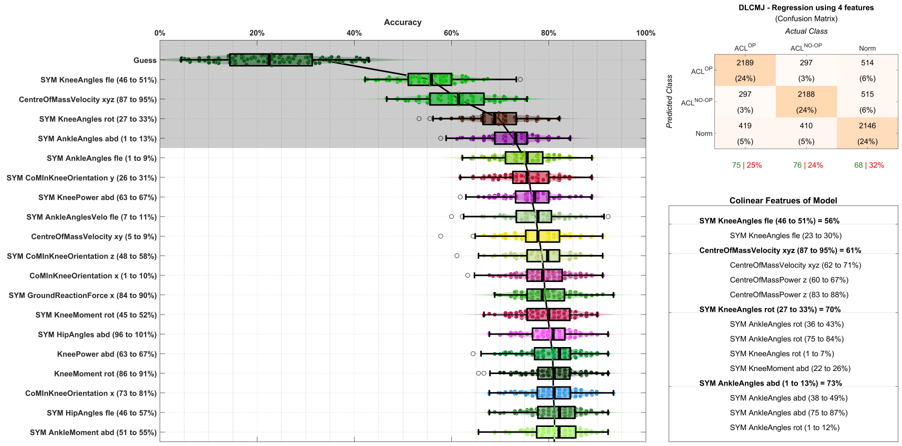

Figure 2: Illustration of the DLCMJ. results. The left side displays the accuracy of the model and the utilised features. The top right displays the confusion matrix of the model using the grey shaded features. Button right reports the selected features and co-linear features.

Selected features in the SLDJ model were: resultant CoM velocity $^{(94\text{-}100\ \%)}$ and vertical CoM velocity $^{(1\text{ to }7\%)}$. The discriminant analysis model with this two features achieved an average accuracy of 56 %. A detailed illustration of the findings is displayed in figure 3.

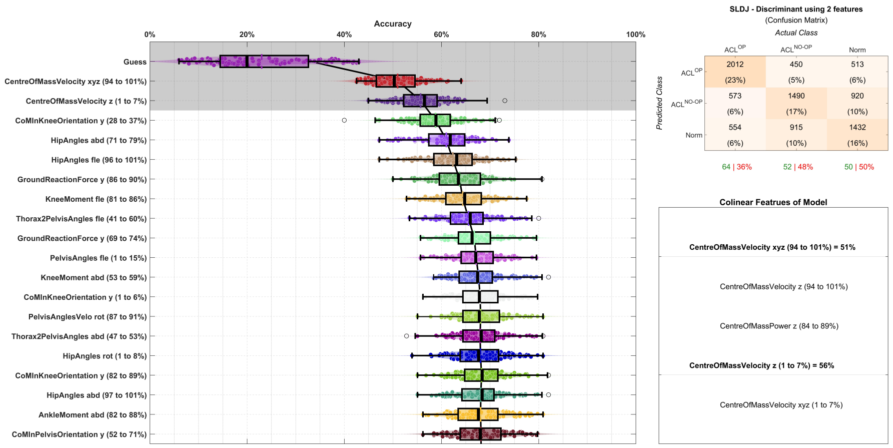

Figure 3: Illustration of the SLDJ results. The left side displays the accuracy of the model and the utilised features. The top right displays the confusion matrix of the model using the grey shaded features. Button right reports the selected features and co-linear features.

Selected features in the DLDJ model were: vertical ground reaction force symmetry $^{(80 \text{ to } 86\ \%)}$, resultant CoM velocity $^{(80 \text{ to } 84\ \%)}$, knee flexion angular velocity $^{(74 \text{ to } 81\ \%)}$. The neural network model with this three features achieved an average accuracy of 81 %. A detailed illustration of the findings is displayed in figure 4.

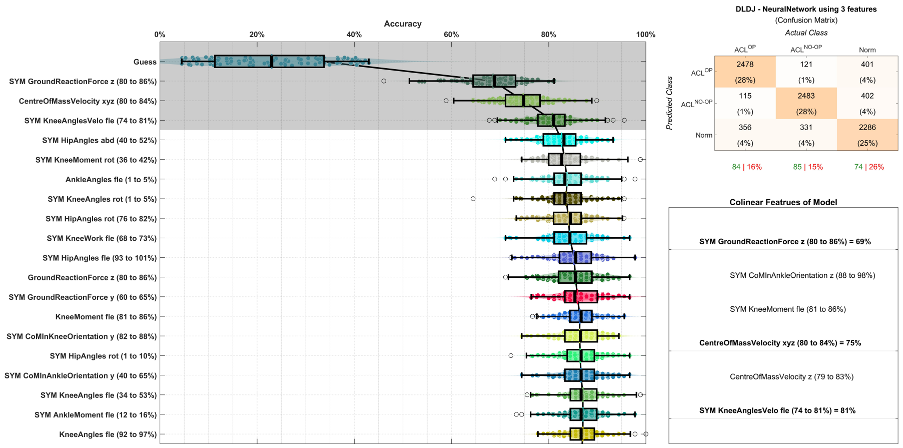

Figure 4: Illustration of the DLDJ results. The left side displays the accuracy of the model and the utilised features. The top right displays the confusion matrix of the model using the grey shaded features. Button right reports the selected features and co-linear features.

Selected features in the SLHop model were: ankle flexion angular velocity $^{(7\text{ to }11\,\%)}$, CoM in knee(transversal plane) $^{(89\text{ to }100\,\%)}$, vertical CoM velocity $^{(1\text{ to }7\,\%)}$, vertical ground reaction force $^{(14\text{ to }19\,\%)}$ and pelvic rotation angles $^{(42\text{ to }50\,\%)}$. The logistic regression model with this five features achieved an average accuracy of 75 %. A detailed illustration of the findings is displayed in figure 5.

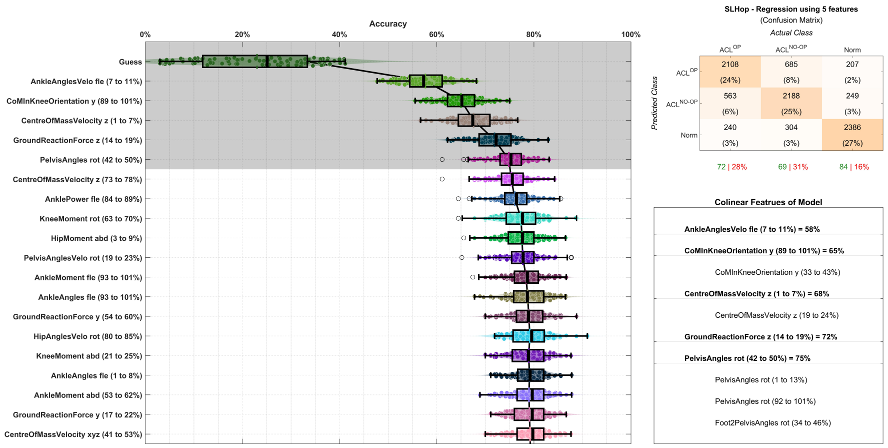

Figure 5: Illustration of the SLHop results. The left side displays the accuracy of the model and the utilised features. The top right displays the confusion matrix of the model using the grey shaded features. Button right reports the selected features and co-linear features.

Selected features in the HuHo model were: knee flexion work $^{(67 \text{ to } 71\,\%)}$, vertical ground reaction force $^{(76 \text{ to } 80\,\%)}$, knee abduction moment $^{(38 \text{ to } 42\,\%)}$, vertical CoM velocity $^{(66 \text{ to } 76\,\%)}$ and knee flexion angular velocity $^{(84 \text{ to } 89\,\%)}$. The logistic regression model with this five features achieved an average accuracy of 69 %. A detailed illustration of the findings is displayed in figure 6.

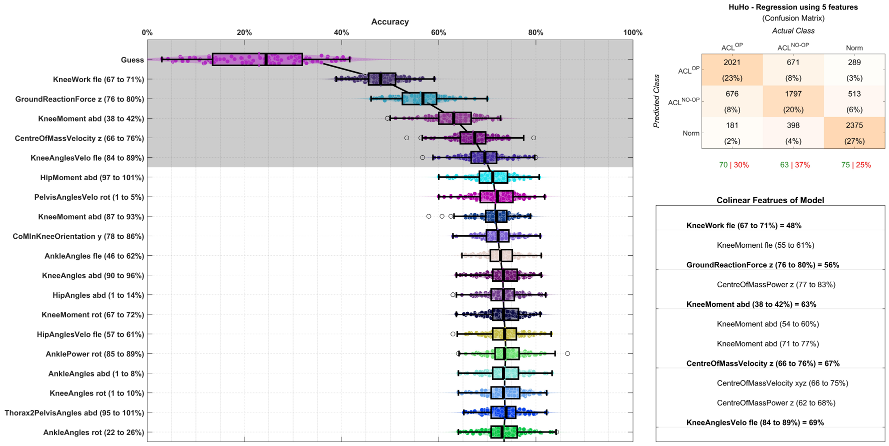

Figure 6: Illustration of the HuHo results. The left side displays the accuracy of the model and the utilised features. The top right displays the confusion matrix of the model using the grey shaded features. Button right reports the selected features and co-linear features.

Selected features in the CoDP model were: knee flexion angular velocity $^{(96\text{ to }100\ \%)}$, knee flexion angle $^{(57\text{ to }61\ \%)}$ and knee abduction moment $^{(52\text{ to }56\ \%)}$. The discriminant analysis model with this three features achieved an average accuracy of 63 %. A detailed illustration of the findings is displayed in figure 7.

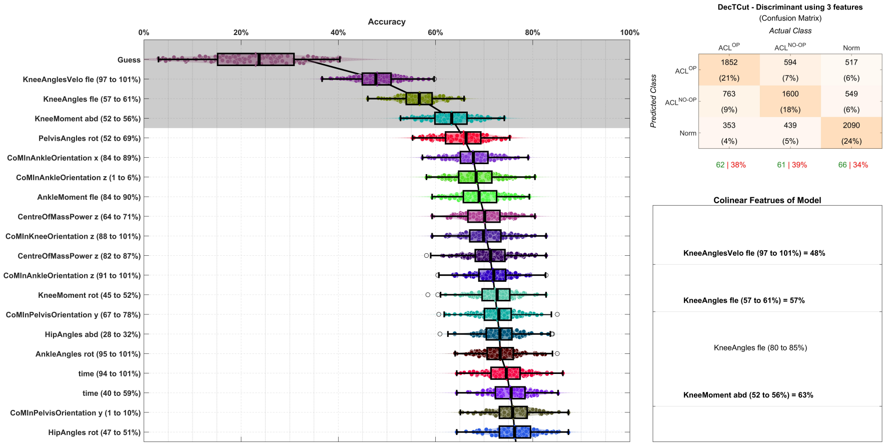

Figure 7: Illustration of the CoDP results. The left side displays the accuracy of the model and the utilised features. The top right displays the confusion matrix of the model using the grey shaded features. Button right reports the selected features and co-linear features.

Selected features in the CoDU model were: vertical CoM velocity $^{(86\text{ to }91\ \%)}$ and hip flexion moment $^{(91\text{ to }95\ \%)}$. The discriminant analysis model with this three features achieved an average accuracy of 52 %. A detailed illustration of the findings is displayed in figure 8.

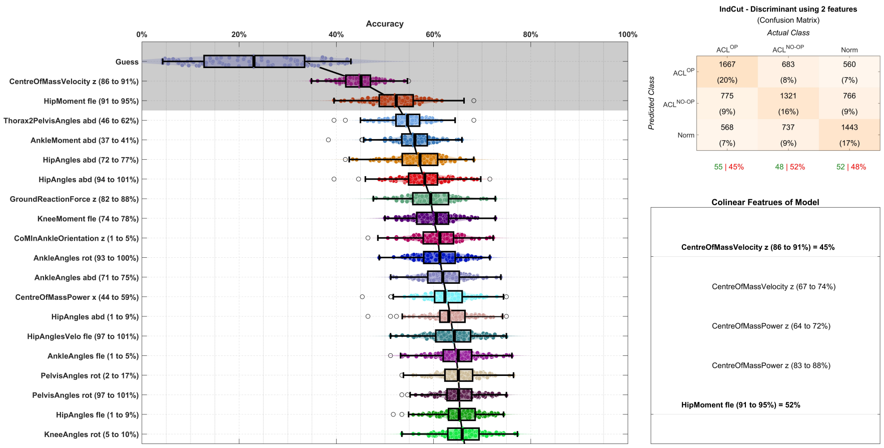

Figure 8: Illustration of CoDU results. The left side displays the accuracy of the model and the utilised features. The top right displays the confusion matrix of the model using the grey shaded features. Button right reports the selected features and co-linear features.
