# Supplementary material for: Objective classification and scoring of movement deficiencies in patients with anterior cruciate ligament reconstruction
Source: PLoS One. 2019 Jul 23;14(7):e0206024. doi: 10.1371/journal.pone.0206024 (PMC6650047; doi:10.1371/journal.pone.0206024)
Supplement: S4 Appendix — (PDF) [file pone.0206024.s004.pdf]

Appendix D - Model Evaluation Selection

This appendix illustrates in detail the evaluation of every model build from the examined exercises.

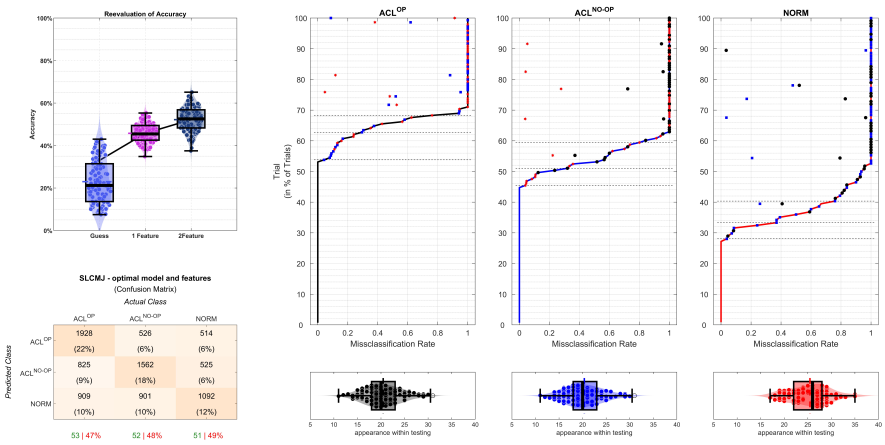

Figure 1: Illustration of the SLCMJ.

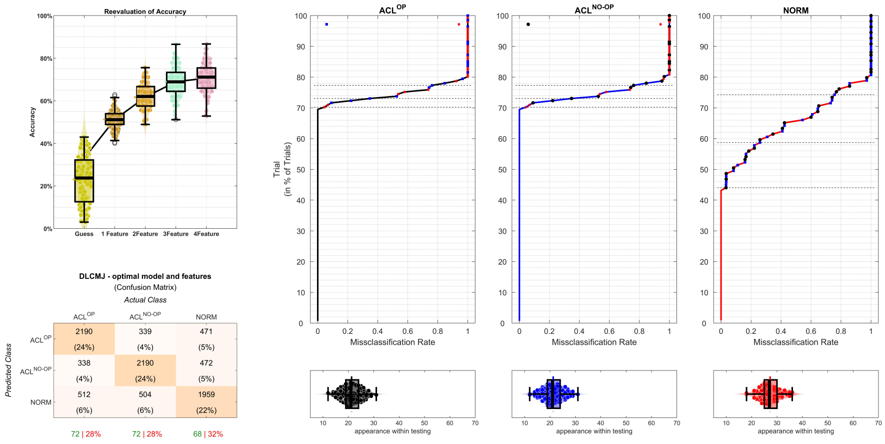

Figure 2: Illustration of the DLCMJ.

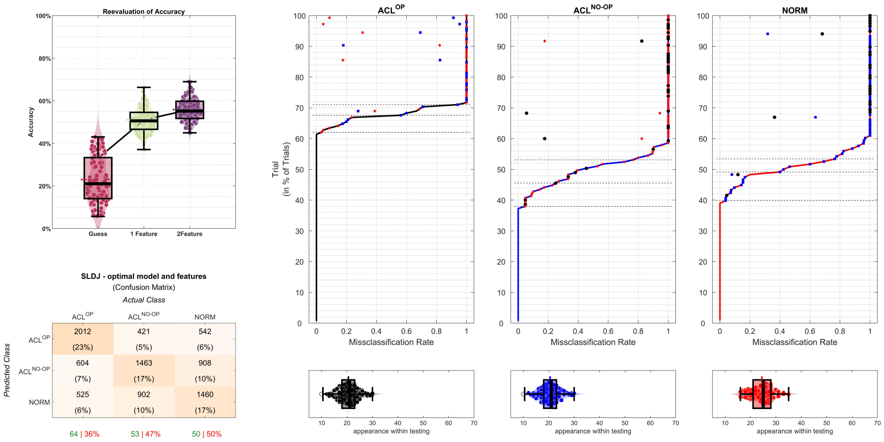

Figure 3: Illustration of the SLDJ.

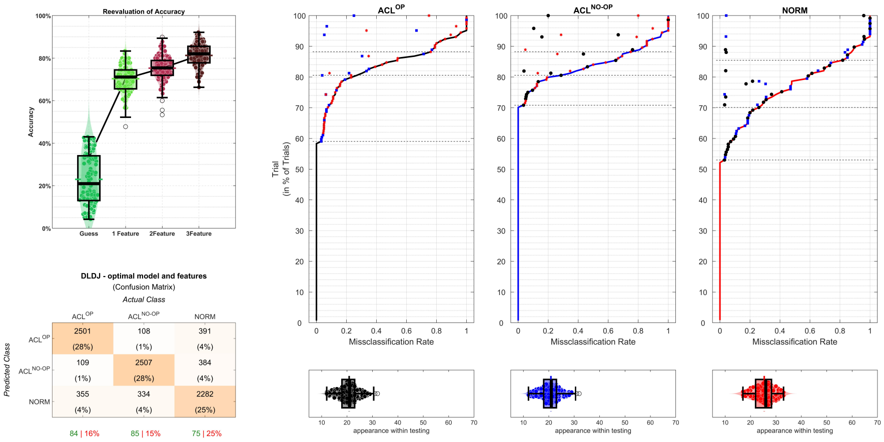

Figure 4: Illustration of the DLDJ.

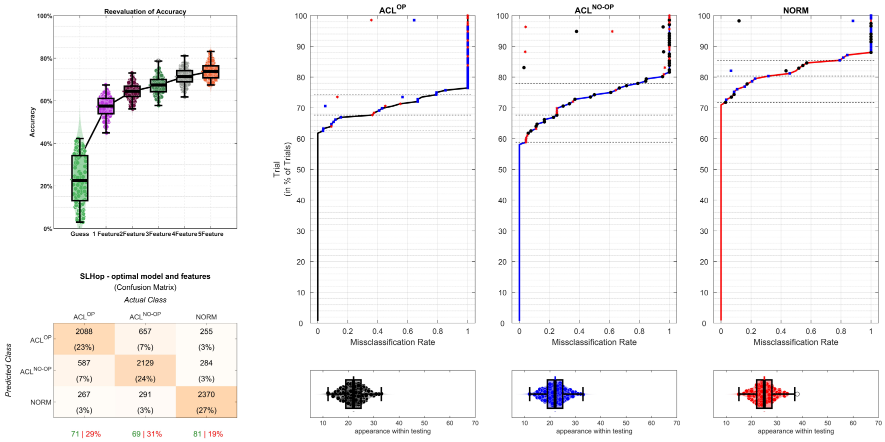

Figure 5: Illustration of the SLHop.

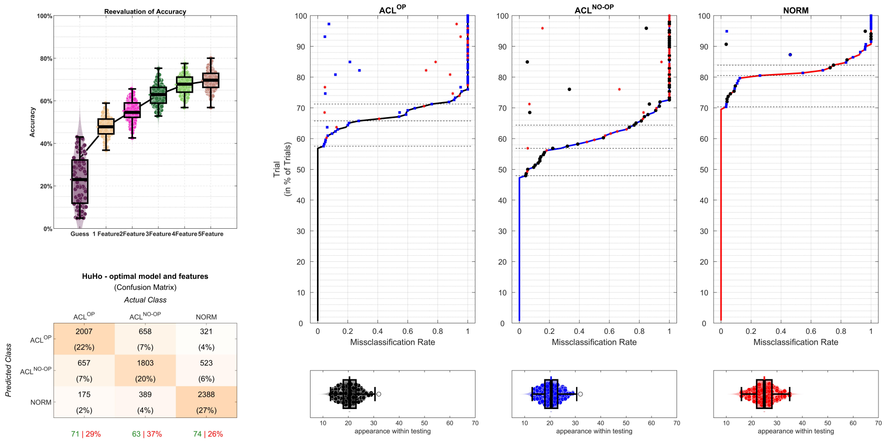

Figure 6: Illustration of the HuHo.

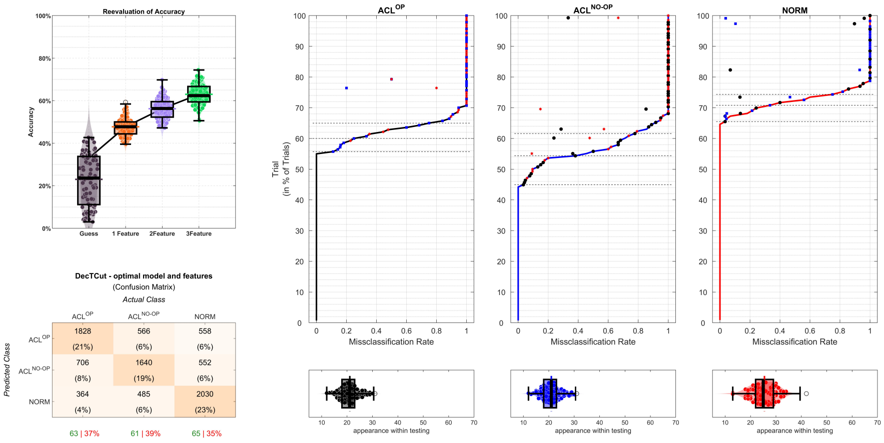

Figure 7: Illustration of the CoDP.

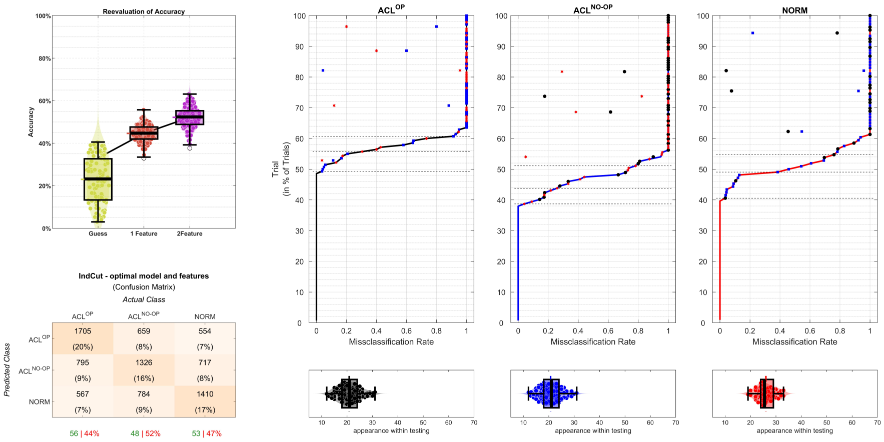

Figure 8: Illustration of CoDU.
